# Protocol for simultaneous detection of mRNAs and (phospho-)proteins with ARTseq-FISH in mouse embryonic stem cells

**DOI:** 10.1016/j.xpro.2024.103336

**Published:** 2024-10-01

**Authors:** Xinyu Hu, Bob van Sluijs, Óscar García-Blay, Wilhelm T.S. Huck, Maike M.K. Hansen

**Affiliations:** 1 Institute for Molecules and Materials, Radboud University, Heyendaalseweg 135, 6525 AJ Nijmegen, the Netherlands; 2 Oncode Institute, Nijmegen, the Netherlands

**Keywords:** Single Cell, Genomics, RNA-seq, Molecular Biology, Gene Expression, Antibody, *In Situ* Hybridization

## Abstract

Understanding the molecular signatures of individual cells within complex biological systems is crucial for deciphering cellular heterogeneity and uncovering regulatory mechanisms. Here, we present a protocol for simultaneous multiplexed detection of selected mRNAs and (phospho-)proteins in mouse embryonic stem cells using spatial single-cell profiling. We describe steps for employing single-stranded DNA (ssDNA)-labeled antibo'dies, padlock probes, and rolling circle amplification to achieve simultaneous visualization of mRNAs and (phospho-)proteins at subcellular resolution. This protocol has potential application in identifying cells in heterogeneous biological microenvironments.

For complete details on the use and execution of this protocol, please refer to Hu et al.^1^

## Highlights

• Guidelines for probe design and instructions for antibody conjugation
• Optimized protocol for combining the hybridization of selected mRNAs and proteins
• Image-based multiplexed detection of mRNAs and (phospho-)proteins in single cells
• Application of ARTseq-FISH for other cell lines and detection approaches (FACS)

## Abstract (editor highlights)

Publisher’s note: Undertaking any experimental protocol requires adherence to local institutional guidelines for laboratory safety and ethics.

## Graphical abstract

**Figure:**
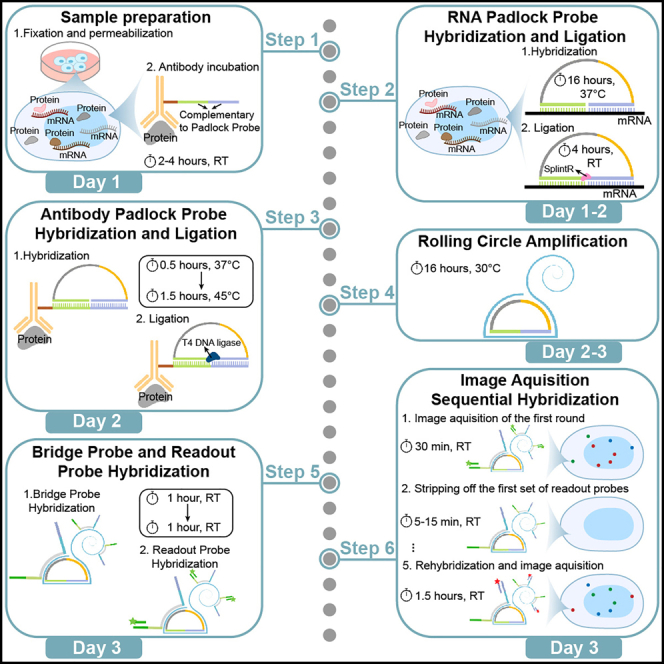

## Before you begin

The protocol below describes the steps for simultaneously profiling of message ribonucleic acid (mRNAs), proteins, and phosphoproteins in individual mouse embryonic stem cells (mESCs). The method is antibody and mRNA targeting sequential fluorescence *in situ* hybridization (ARTseq-FISH). ARTseq-FISH detects mRNAs and proteins at the same resolution and is scalable, which enables analysis without the need to integrate transcriptomic and proteomic data.

ARTseq-FISH requires antibodies conjugated with a single stranded deoxyribonucleic acid (ssDNA) sequence, a series of ssDNA probes, and enzymatic reactions. In this protocol, we describe the antibody conjugation, design of the probes, step-by-step procedure of ARTseq-FISH, and the visualization of the detected signals. We optimized ARTseq-FISH on mESCs and provide the specific workflow of performing ARTseq-FISH on mESCs to quantify 67 mRNAs and (phospho-)proteins in micropatterned mESCs during the exit of pluripotency.^1^ Furthermore, this technique was partially validated in MCF7 and 3T3 cells and we expect it to be readily adaptable to any cell-type or tissue type. We visualized the detected signals either by microscopy or flow cytometry.

### Institutional permissions

No experiments were performed on live vertebrates or higher invertebrates - institutional permissions are not required.

### Probe design

**Timing: 1–2 days, variable depending on the number of targets selected**

**Figure 1:**
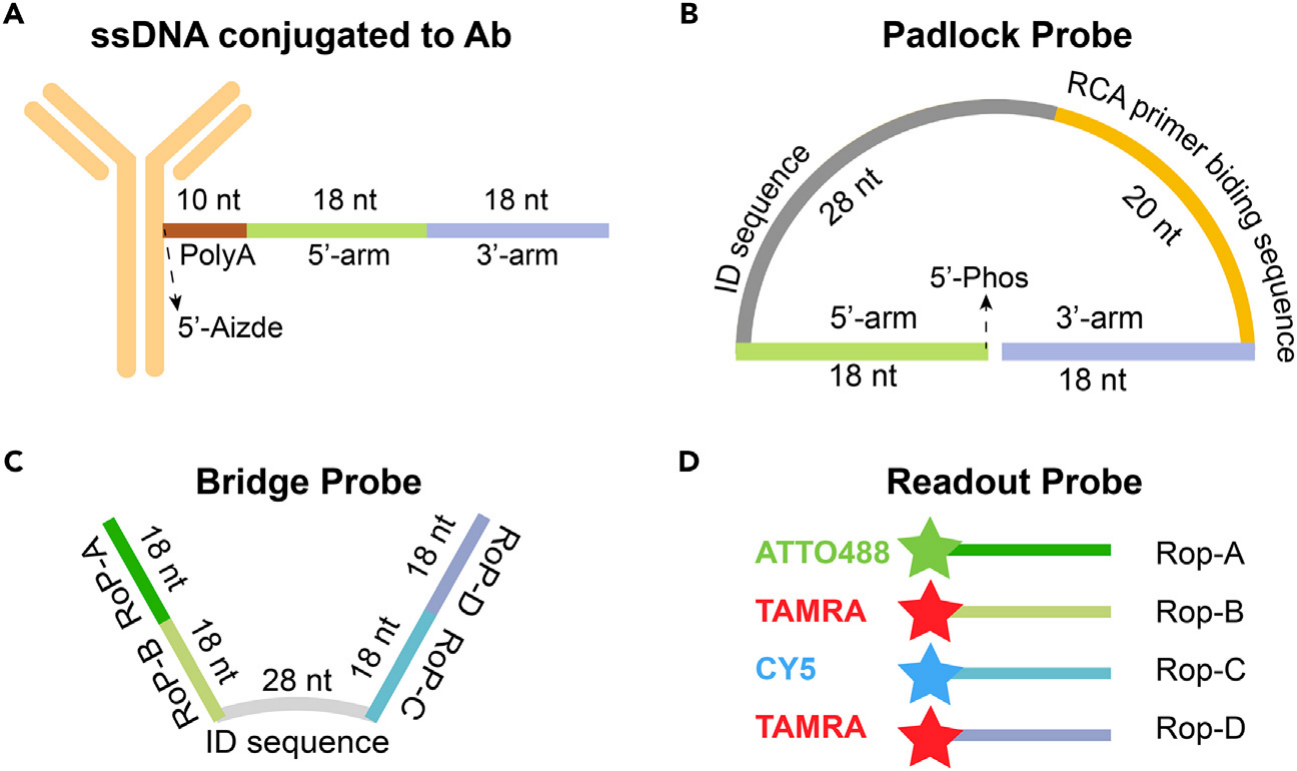
Schematics of ssDNA tagged antibody and probes in ARTseq-FISH (A) The ssDNA labeled to antibodies are designed as 10 nt polyadenylate A (Poly A) and two 18 nt sequences (purple and light-yellow lines) complementary to unique PLPs. (B) PLPs consist of two 18 nt sequences (purple and light-yellow lines) recognizing the ssDNA of protein targets or cellular mRNAs, a 28 nt ID sequence (black line) for corresponding to unique BrP, and a 20 nt RCA primer binding sequence. (C) BrPs are composed of a 28 nt ID sequence (black line) identical to the ID sequence of PLPs, four 18 nt sequences for different readout probes binding. (D) RoPs are an 18 nt single strand DNA conjugated with three different fluorophores (ATTO488, TAMRA and CY5).

In ARTseq-FISH, we make use of the color barcode scheme described in previous work^2^^,^^3^^,^^4^^,^^5^ to scale up the multiplexing capacity. To combine the protocol of sequential mRNA and protein detection via FISH, we utilized ssDNA-labeled antibodies (Figure 1A), and padlock probes (PLPs) (Figure 1B). The limited length of the ssDNA sequence conjugated to antibodies^6^ requires an amplification step, which is also necessary to combine the detection of mRNA and protein. Therefore, we adopted the rolling circle amplification (RCA) of PLPs as previous reported.^7^ To this end, we designed a unique 36 nucleotides (nt) ssDNA oligonucleotide with a 10 nt polyadenylate linker for each protein target (Table 1). For mRNA targets, the PLPs are directly complementary to a 36 nt targeting region on the endogenous mRNA. On each PLP, there are one target-specific identical (ID)-sequence of 28 nt and one universal RCA primer binding region of 20 nt. Two successive different ligation reactions will seal the respective gaps between the 5′- and 3′-arms of the mRNA PLPs and the antibody PLPs. After PLPs circularization (through ligation), the RCA reaction will amplify the sequence of the PLPs by generating a long amplification product that contains a repetitive complementary sequence of each PLP. Each target-specific bridge probe (BrP) can hybridize to the ID-sequence from their respective PLP, followed by the hybridization of the readout probe (RoP) to the BrP in a target-specific combination throughout the hybridization rounds (Figures 1B–1D; Tables 1, 2, and 3). Each target is associated with four different RoPs labeled with three different fluorophores (i.e., ATTO 488, TAMRA, and CY5). These fluorophores were chosen based on the available fluorescent channels in our spinning disc confocal microscope. During sequential hybridization, each color (i.e., channel of the fluorophores) represents multiple targets. For example, in our experiment,^1^ one color represented up to 16 targets in a single hybridization round. We distinguish the targets through a barcoding system that combines the sequence of hybridization rounds with the corresponding fluorophore colors. Specifically, target 1 has a barcode of R (Red), B (Blue), G (Green), R (Red). This means that if a spot appears as a red dot in the first round, blue in the second round, green in the third round, and red in the fourth round, then this spot corresponds to target 1.

**Table 1:** The component of antibody-tagging ssDNA, padlock probes, bridge probe and readout probes

Antibody-tagging ssDNA (46 nt)	5′- azide-AAAAAAAAAA- [36 nt unique sequence]-3′
Padlock probe (84 nt)	5′ -phosphorylated- [18 nt 5′-arm] - [28 nt unique sequence] - [20 nt RCA primer] - [18 nt 3′- arm] - 3′
Bridge probe (108 nt)	5′ - [readout 1] -AA- [readout 2] -AA- [28 nt unique sequence] -AA- [readout 3] -AA- [readout 4] - 3′
Readout probe (18 nt)	5′-fluorophores- [18 nt sequence]

The structure of each probe used in ARTseq-FISH. For ssDNA, we designed an azide modification on the 5′ end, followed by 10 nt polyA and the 36 nt target specific sequences. All PLPs are equipped with a 5′-phosphate at the 5′ end, followed by 18 nt 5′ arm, 28 nt ID sequence (identical to part of the BrPs sequence), 20 nt RCA primer binding sequence, and 18 nt 3′arm. For BrPs, there are four different regions for RoPs to bind and two AA in between. For RoPs, there are three fluorophores conjugated to different RoPs.

**Table 2:** Sequences of readout probes

	Ordering code	Sequence	Ex/Em	Fluorophore
1	R1-Tm1	GGA GCG AGA GAC CAT AGC	546/579	TAMRA
2	G1-AT1	GGT TGA ACG CAA TGC CGC	502/522	ATTO488
3	FR1-CY1	GGA ATT GAC CGA CCG TGG	647/668	CY5
4	R2-Tm2	TTA AGC GCC CCG TAC CTA	546/579	TAMRA
5	G2-AT2	GCT GTT ACG CGC TCG GTT	502/522	ATTO488
6	FR2-CY2	CCG CTA CAC GAT CGT ACT	647/668	CY5
7	R3-Tm3	TTA CGA GCG CTT GGA TCC	546/579	TAMRA
8	G3-AT3	CTT AAC CGA ACT GAC GGC	502/522	ATTO488
9	FR3-CY3	CTA TTC TAA GCC GGC GGT	647/668	CY5
10	R4-Tm4	ATG AGG ACG AAT CTC CCG	546/579	TAMRA
11	G4-AT4	TGT ACC GTT TAT CGG GCG	502/522	ATTO488
12	FR4-CY5	TGC TCG CAT ACC CGA TGT	645/665	CY5
13	R_d-TmD1	GCC GCT AGA AAG ACC TCG	559/583	TAMRA
14	R_d-TmD2	GTC CCG ACA GTG GTT AAC	559/583	TAMRA

The sequences and fluorophores for each RoP used in ARTseq-FISH.

**Table 3:** Probe sequences in ARTseq-FISH

	Sequence
**Smad1_Ab**	

Smad1_Ab_tag	AAA AAA AAA A GGA ATG CTT GTG ACG TGC GCA CTC AAC GCG TTC AGC
Smad1_Ab_PLP	GCACGTCACAAGCATTCCGCT ATT CAC CGC GTT ACT CTT CGT CCG CTGCGTCTATTTAGTG GAGCCGCTGAACGCGTTGAGTGC
Smad1_Ab_BrP	GCT ATG GTC TCT CGC TCC AA GCG GCA TTG CGT TCA ACC AA GCT ATT CAC CGC GTT ACT CTT CGT CCG CAA CCA CGG TCG GTC AAT TCC AA CGA GGT CTT TCT AGC GGC

**Nanog_Ab**	

Nanog_Ab_tag	AAA AAA AAA A ATG CAC GGT GCG CGT TAG GGG GAT GTC CCG ACA ATA
Nanog_Ab_PLP	CTAACGCGCACCGTGCATTGG GAC CGG TTC ATT GTG GCG CGA CTC GTGCGTCTATTTAGTG GAGCCTATTGTCGGGACATCCCC
Nanog_Ab_BrP	GGA TCC AAG CGC TCG TAA AA GCC GTC AGT TCG GTT AAG AA TGG GAC CGG TTC ATT GTG GCG CGA CTC G AA ACC GCC GGC TTA GAA TAG AA CGA GGT CTT TCT AGC GGC

**pSmad1_Ab**	

pSmad1/5_Ab_tag	AAA AAA AAA A CCG TTG CTA ACT CGA CTG TCG GCC GGT AAT GGT ATG
pSmad1/5_Ab_PLP	CAGTCGAGTTAGCAACGG CTCGTGACGGTGCCGGCTCTGCACAATG TGCGTCTATTTAGTGGAG CCCATACCATTACCGGCCGA
pSmad1/5_Ab_BrP	GCT ATG GTC TCT CGC TCC AA GCG GCA TTG CGT TCA ACC AA CTC GTG ACG GTG CCG GCT CTG CAC AAT GAA ACC GCC GGC TTA GAA TAG AA CGA GGT CTT TCT AGC GGC

**SRSF3_Ab**	

SRSF3_Ab_tag	AAA AAA AAA ACGT CTA CAT CGC GAG CTT GTC ATA TGA AGC GCT GGG
SRSF3_Ab_PLP	AAGCTCGCGATGTAGACGGACTAGGAAGCTAGAGCCAGGCGCGACGTGCGTCTATTTAGTGGAG CCCCCAGCGCTTCATATGAC
SRSF3_Ab_BrP	TAG GTA CGG GGC GCT TAA AA GCG GCA TTG CGT TCA ACC AA GAC TAG GAA GCT AGA GCC AGG CGC GAC GAA ACC GCC GGC TTA GAA TAG AA CGA GGT CTT TCT AGC GGC

**OCT4_Ab**	

OCT4_Ab_tag	AAA AAA AAA AAGT CGA TTG TCG CTC GTG ATT CGT CGC GGA CAA GGG
OCT4_Ab_PLP	CACGAGCGACAATCGACTGGCGCAAGTAGACATTAGGCTCGGTGGCTGCGTCTATTTAGTGGAGC CCCCTTGTCCGCGACGAAT
OCT4_Ab_BrP	CGG GAG ATT CGT CCT CAT AA AAC CGA GCG CGT AAC AGC AA GGC GCA AGT AGA CAT TAG GCT CGG TGG CAA CCA CGG TCG GTC AAT TCC AA CGA GGT CTT TCT AGC GGC

**TCF1_Ab**	

TCF1_Ab_tag	AAA AAA AAA ACGC GCA CGA ATC GTA TCT CTC ACG GAC TTA GGC ATG
TCF1_Ab_PLP	AGATACGATTCGTGCGCGGTTTCGCCCACGGTTGGATCCAGCTCCTTGCGTCTATTTAGTGGAGC CCATGCCTAAGTCCGTGAG
TCF1_Ab_BrP	CGG GAG ATT CGT CCT CAT AA GCG GCA TTG CGT TCA ACC AA GTT TCG CCC ACG GTT GGA TCC AGC TCC TAA CCA CGG TCG GTC AAT TCC AA CGA GGT CTT TCT AGC GGC

**TCF3_Ab**	

TCF3_Ab_tag	AAA AAA AAA AAGT TGA ACG CAA TGC CGC GCC GCG AGA AAG ACC TAG
TCF3_Ab_PLP	GCGGCATTGCGTTCAACTAGATGGAGAGTGCAGGACATACTCTCGTTGCGTCTATTTAGTGGAGCC CTAGGTCTTTCTCGCGGC
TCF3_Ab_BrP	GGA TCC AAG CGC TCG TAA AA AAC CGA GCG CGT AAC AGC AA AGA TGG AGA GTG CAG GAC ATA CTC TCG TAA ACC GCC GGC TTA GAA TAG AA CGA GGT CTT TCT AGC GGC

**RB_Ab**	

RB_Ab_tag	AAA AAA AAA AACG TCC TTG TCG ACA TGC ACG GAC GTA TGG CGG ACA
RB_Ab_PLP	GCATGTCGACAAGGACGTAGAATACGTCACCACAACTCGGACGTGATGCGTCTATTTAGTGGAGCC TGTCCGCCATACGTCCGT
RB_Ab_BrP	TAG GTA CGG GGC GCT TAA AA AAC CGA GCG CGT AAC AGC AA AGA ATA CGT CAC CAC AAC TCG GAC GTG AAA ACC GCC GGC TTA GAA TAG AA CGA GGT CTT TCT AGC GGC

**Phos-RB_Ab**	

Phos-RB_Ab_tag	AAA AAA AAA AGGC GAA CGG ACC GAA GTA AGA CGA CGG GCT AGG TAG
Phos-RB_Ab_PLP	TACTTCGGTCCGTTCGCCGCACACTATGAAGCACAGCACGCAATCCTGCGTCTATTTAGTGGAGCC CTACCTAGCCCGTCGTCT
Phos-RB_Ab_BrP	CGG GAG ATT CGT CCT CATAA GCC GTC AGT TCG GTT AAG AA GCA CAC TAT GAA GCA CAG CAC GCA ATC CAA CCA CGG TCG GTC AAT TCC AA CGA GGT CTT TCT AGC GGC

**p21_Ab**	

p21_Ab_tag	AAA AAA AAA AAGG TAA ACG AGT AGG CCC GTA GGC GTG CCT AGA AGA
p21_Ab_PLP	GGGCCTACTCGTTTACCTCGCTCATGCGAACTTGGTTGTATCCTGCTGCGTCTATTTAGTGGAG CCTCTTCTAGGCACGCCTAC
p21_Ab_BrP	GGA TCC AAG CGC TCG TAA AA AAC CGA GCG CGT AAC AGC AA CGCTCATGCGAACTTGGTTG TATCCTGC AA ACA TCG GGT ATG CGA GCA AA CGA GGT CTT TCT AGC GGC

**p53_Ab**	

p53_Ab_tag	AAA AAA AAA ACCC GTC GAG GTA TCG AAT ACC GAT AAC GCA CGA GCT
p53_Ab_PLP	ATTCGATACCTCGACGGGACA GCT GCG TGA TCA CTC GAA CTA GCA GTGCGTCTATTTAGTG GAGCCAGCTCGTGCGTTATCGGT
p53_Ab_BrP	GCT ATG GTC TCT CGC TCC AA GCC GTC AGT TCG GTT AAG AA ACA GCT GCG TGA TCA CTC GAA CTA GCA G AA CCACGGTCGGTCAATTCC AA CGA GGT CTT TCT AGC GGC

**Cyclin D_Ab**	

Cyclin D_Ab_tag	AAA AAA AAA AGCG GAA CGG CAA TCA TGT CAT ATT GCG GAG AGT CGC
Cyclin D_Ab_PLP	ACATGATTGCCGTTCCGCATG GCA GTC AGA CAT GTC TGT ACT GCC ATGCGTCTATTTAGTG GAGCCGCGACTCTCCGCAATATG
Cyclin D_Ab_BrP	GCT ATG GTC TCT CGC TCC AA GCC GTC AGT TCG GTT AAG AA ATG GCA GTC AGA CAT GTC TGT ACT GCCA AA ACC GCC GGC TTA GAA TAG AA CGA GGT CTT TCT AGC GGC

**CDK4_Ab**	

CDK4_Ab_tag	AAA AAA AAA ATAC TAC GTT CGA GCG GCG GGC GTA TCA CGA CCG TTA
CDK4_Ab_PLP	CGCCGCTCGAACGTAGTACTT ATG CGA CAT GGT TGG CGG ACA ATC CTGCGTCTATTTAGTG GAGCCTAACGGTCGTGATACGCC
CDK4_Ab_BrP	CGG GAG ATT CGT CCT CAT AA GCG GCA TTG CGT TCA ACC AA CTT ATG CGA CAT GGT TGG CGG ACA ATC C AA AGT ACG ATC GTG TAG CGG AA CGA GGT CTT TCT AGC GGC

**CDK2_Ab**	

CDK2_Ab_tag	AAA AAA AAA ATCG CCG GTA CGA AGA GTA ATG TTT GCG CGG GAC TTC
CDK2_Ab_PLP	TACTCTTCGTACCGGCGATGT CCT GTC CTG TAC GAA ATC ATT GGT CTGCGTCTATTTAGTG GAGCCGAAGTCCCGCGCAAACAT
CDK2_Ab_BrP	TAG GTA CGG GGC GCT TAA AA GCG GCA TTG CGT TCA ACC AA TGT CCT GTC CTG TAC GAA ATC ATT GGT C AA ACA TCG GGT ATG CGA GCA AA CGA GGT CTT TCT AGC GGC

**Cyclin E_Ab**	

Cyclin E_Ab_tag	AAA AAA AAA AGGT AAT GTC GCG ACG GTC GTC TCG TCA AGT CGT TGC
Cyclin E_Ab_PLP	GACCGTCGCGACATTACCTGA CTG AAC CAG ATC CTC CGA TGT TCT TTGCGTCTATTTAGT GGAGCCGCAACGACTTGACGAGAC
Cyclin E_Ab_BrP	TAG GTA CGG GGC GCT TAA AA GCC GTC AGT TCG GTT AAG AA TGA CTG AAC CAG ATC CTC CGA TGT TCT T AA AGT ACG ATC GTG TAG CGG AA CGA GGT CTT TCT AGC GGC

**CDKN2A (P16Ink4a) _Ab**	

CDKN2A (P16Ink4a) _Ab_tag	AAA AAA AAA AACG ATA CGC CAA TCG GGA CGG GGG TCA TCG CGT AAA
CDKN2A (P16Ink4a) _Ab_PLP	TCCCGATTGGCGTATCGTACC GAC ATG TGG TGC CAC AGA GTC ATT CTGCGTCTATTTAG TGGAGCCTTTACGCGATGACCCCCG
CDKN2A (P16Ink4a) _Ab_BrP	TAG GTA CGG GGC GCT TAA AA GCC GTC AGT TCG GTT AAG AA ACC GAC ATG TGG TGC CAC AGA GTC ATT C AA CCACGGTCGGTCAATTCC AA CGA GGT CTT TCT AGC GGC

**Phos-Pol II_Ab**	

Phos-Pol II_Ab_tag	AAA AAA AAA AGCG CAC GTT ATG AAC GCG GCG AAC CGA CGA TAC CGA
Phos-Pol II_Ab_PLP	CGCGTTCATAACGTGCGCAGA CAT GGT GCT TAG GGC GAT TCA TCA GTGCGTCTATTTA GTGGAGCCTCGGTATCGTCGGTTCGC
Phos-Pol II_Ab_BrP	GCT ATG GTC TCT CGC TCC AA GCC GTC AGT TCG GTT AAG AA AGA CAT GGT GCT TAG GGC GAT TCA TCA GAA AGT ACG ATC GTG TAG CGG AA CGA GGT CTT TCT AGC GGC

**STAT3_Ab**	

STAT3_Ab_tag	AAA AAA AAA AGCT GAT AAG TCG CGC ATT CGG TCT ACC GAT CGT AGA
STAT3_Ab_PLP	AATGCGCGACTTATCAGCAGA GTG ACA TGC TCT AAC TGA CCG TAT GTGCGTCTATTTA GTGGAGCCTCTACGATCGGTAGACCG
STAT3_Ab_BrP	GCT ATG GTC TCT CGC TCC AA AAC CGA GCG CGT AAC AGC AA AGA GTG ACA TGC TCT AAC TGA CCG TAT GAA ACA TCG GGT ATG CGA GCA AA CGA GGT CTT TCT AGC GGC

**Phos-STAT3_Ab**	

Phos-STAT3_Ab_tag	AAA AAA AAA AAGC TCG AGC GAT ACC ACG GTA TCG TGA CGG CTA GGA
Phos-STAT3_Ab_PLP	CGTGGTATCGCTCGAGCTGAG CAC GCC TAG ACA AGT GAG TTA ACT CTGCGTCTATTTAGT GGAGCCTCCTAGCCGTCACGATAC
Phos-STAT3_Ab_BrP	TAG GTA CGG GGC GCT TAA AA GCG GCA TTG CGT TCA ACC AA GAG CAC GCC TAG ACA AGT GAG TTA ACT C AA AGT ACG ATC GTG TAG CGG AA CGA GGT CTT TCT AGC GGC

**AKT_Ab**	

AKT_Ab_tag	AAA AAA AAA AACG ATA ATG ACC CGC GGA GGG CAC TAT TGC GCG AAC
AKT_Ab_PLP	TCCGCGGGTCATTATCGTAGT TAA CGT TAC AGC TGC TGG ACG ACT GTGCGTCTATTTAGTG GAGCCGTTCGCGCAATAGTGCCC
AKT_Ab_BrP	GCT ATG GTC TCT CGC TCC AA GCG GCA TTG CGT TCA ACC AA AGT TAA CGT TAC AGC TGC TGG ACG ACT GAA AGT ACG ATC GTG TAG CGG AA CGA GGT CTT TCT AGC GGC

**Phos-AKT_Ab**	

Phos-AKT_Ab_tag	AAA AAA AAA AAGC GCG GTA TAC GTT GTC CGC ACG ACT TAT CAC GCA
Phos-AKT_Ab_PLP	GACAACGTATACCGCGCTTCG TTA CGT TGG TGA GGC TCT TGA GTA CTGCGTCTATTTAGTG GAGCCTGCGTGATAAGTCGTGCG
Phos-AKT_Ab_BrP	CGG GAG ATT CGT CCT CAT AA AAC CGA GCG CGT AAC AGC AA TCG TTA CGT TGG TGA GGC TCT TGA GTA CAA AGT ACG ATC GTG TAG CGG AA CGA GGT CTT TCT AGC GGC

**Smad 2_Ab**	

Smad 2_Ab_tag	AAA AAA AAA AGGA CGG TTC CAA TCG GAT TAC GCG CGC TAT AGT ACC
Smad 2_Ab_PLP	ATCCGATTGGAACCGTCCGTT CAT TGA TCG GTG TCG CGA GTA GAC TTGCGTCTATTTAGTGG AGCCGGTACTATAGCGCGCGTA
Smad 2_Ab_BrP	GGA TCC AAG CGC TCG TAA AA AAC CGA GCG CGT AAC AGC AA GTT CAT TGA TCG GTG TCG CGA GTA GAC TAA AGT ACG ATC GTG TAG CGG AA CGA GGT CTT TCT AGC GGC

**Phos-Smad2_Ab**	

Phos-Smad2_Ab_tag	AAA AAA AAA AGCG TCG TAG AAC GAG TCC GTA CGG CTA GCT CAA CGG
Phos-Smad2_Ab_PLP	GGACTCGTTCTACGACGCAGA TGA TGA TAT CGA CGA GTC TTG CCT CTGCGTCTATTTAGTGG AGCCCCGTTGAGCTAGCCGTAC
Phos-Smad2_Ab_BrP	GGA TCC AAG CGC TCG TAA AA GCG GCA TTG CGT TCA ACC AA AGA TGA TGA TAT CGA CGA GTC TTG CCT CAA ACA TCG GGT ATG CGA GCA AA CGA GGT CTT TCT AGC GGC

**Beta-Catenin_Ab**	

Beta-Catenin_Ab_tag	AAA AAA AAA ATCC GCG ATA TTC GCA TGA TGC AAA CTC GCC GCG TAA
Beta-Catenin_Ab_PLP	TCATGCGAATATCGCGGATGG TGA CTG GAT GGG TGG TCA TGA TCT CTGCGTCTATTTAGTGGA GCCTTACGCGGCGAGTTTGCA
Beta-Catenin_Ab_BrP	TAG GTA CGG GGC GCT TAA AA AAC CGA GCG CGT AAC AGC AA TGG TGA CTG GAT GGG TGG TCA TGA TCT CAA CCACGGTCGGTCAATTCC AA CGA GGT CTT TCT AGC GGC

**Phos-beta-catenin_Ab**	

Phos-beta-catenin_Ab_tag	AAA AAA AAA ACGT GTC GCG ACC GAC AAG GTC ACG TTG GGC GAA CTT
Phos-beta-catenin_Ab_PLP	CTTGTCGGTCGCGACACGAGA GTG AAG GAC CTG TCG TCG TAG CAC TTGCGTCTATTTAGTGGA GCCAAGTTCGCCCAACGTGAC
Phos-beta-catenin_Ab_BrP	GCT ATG GTC TCT CGC TCC AA AAC CGA GCG CGT AAC AGC AA AGA GTG AAG GAC CTG TCG TCG TAG CAC TAA ACC GCC GGC TTA GAA TAG AA CGA GGT CTT TCT AGC GGC

**E-cadherin_Ab**	

E-cadherin_Ab_tag	AAA AAA AAA AAAG ACC GCC TAT TCG TCA GGC GAC GAC TTC TCG TTA
E-cadherin_Ab_PLP	TGACGAATAGGCGGTCTTTGT ACG ACG ATA GTA CTC GAG GTC CTG TTGCGTCTATTTAGTGGAGC CTAACGAGAAGTCGTCGCC
E-cadherin_Ab_BrP	CGG GAG ATT CGT CCT CAT AA GCG GCA TTG CGT TCA ACC AA TGT ACG ACG ATA GTA CTC GAG GTC CTG T AA ACC GCC GGC TTA GAA TAG AA CGA GGT CTT TCT AGC GGC

**YAP_Ab**	

YAP_Ab_tag	AAA AAA AAA AATT TCT GCG CCG AAT CGG ATC TCC GAT CGA CAG TGC
YAP_Ab_PLP	CCGATTCGGCGCAGAAATGCA AAC CAA GAG GAG CAC TTC ATG CAG CTGCGTCTATTTAGTGGAG CCGCACTGTCGATCGGAGAT
YAP_Ab_BrP	CGG GAG ATT CGT CCT CAT AA GCG GCA TTG CGT TCA ACC AA GCA AAC CAA GAG GAG CAC TTC ATG CAG C AA ACA TCG GGT ATG CGA GCA AA CGA GGT CTT TCT AGC GGC

**Phos-YAP_Ab**	

Phos-YAP_Ab_tag	AAA AAA AAA ATAG ACT GAC CGT CGC GTA GTC ATC GGA CGG TAT TCG
Phos-YAP_Ab_PLP	TACGCGACGGTCAGTCTAGAT CGA CAT GAG TTG TGC AGC AGT GAC GTGCGTCTATTTAGTGGAG CCCGAATACCGTCCGATGAC
Phos-YAP_Ab_BrP	GGA TCC AAG CGC TCG TAA AA AAC CGA GCG CGT AAC AGC AA GAT CGA CAT GAG TTG TGC AGC AGT GAC GAA CCACGGTCGGTCAATTCC AA CGA GGT CTT TCT AGC GGC

**Brachyury_Ab**	

Brachyury_Ab_tag	AAA AAA AAA ACGT ACC CGA ATC GCG TAA TTG GTA GAA TGC GAG GCA
Brachyury_Ab_PLP	TTACGCGATTCGGGTACGACA GCT CCA AGC CTC GTT TGT GAT TGT CTGCGTCTATTTAGTGGAGCC TGCCTCGCATTCTACCAA
Brachyury_Ab_BrP	GGA TCC AAG CGC TCG TAA AA GCG GCA TTG CGT TCA ACC AA ACA GCT CCA AGC CTC GTT TGT GAT TGT CAA AGT ACG ATC GTG TAG CGG AA CGA GGT CTT TCT AGC GGC

**Sox17_Ab**	

Sox17_Ab_tag	AAA AAA AAA ATAG CCC GTC GCG TAT AAC GGA GCG ATG CGG TCA ATC
Sox17_Ab_PLP	GTTATACGCGACGGGCTAAAG ACC TCG TTG TTC GGG CGT CTA CCA TTGCGTCTATTTAGTGGAGCC GATTGACCGCATCGCTCC
Sox17_Ab_BrP	GCT ATG GTC TCT CGC TCC AA GCG GCA TTG CGT TCA ACC AA AAG ACC TCG TTG TTC GGG CGT CTA CCA T AA ACA TCG GGT ATG CGA GCA AA CGA GGT CTT TCT AGC GGC

**GATA6_Ab**	

GATA6_Ab_tag	AAA AAA AAA AATC GAT CGT CGT CGG GAC GCT CGC GTA TAG TTA GCG
GATA6_Ab_PLP	GTCCCGACGACGATCGATTCT TTC AAA GCA CAG CGG GAT GTC ATC GTGCGTCTATTTAGTGGAGCC CGCTAACTATACGCGAGC
GATA6_Ab_BrP	GCT ATG GTC TCT CGC TCC AA AAC CGA GCG CGT AAC AGC AA TCT TTC AAA GCA CAG CGG GAT GTC ATC GAA AGT ACG ATC GTG TAG CGG AA CGA GGT CTT TCT AGC GGC

**Axin2_Ab**	

Axin2_Ab_tag	AAA AAA AAA ACCG CAT CGC GAC ACA ATT CGA TCG CGG AGC GTT TAG
Axin2_Ab_PLP	AATTGTGTCGCGATGCGGGCG GTC TGT ATC CGA ATC TCA CGC TAT ATGCGTCTATTTAGTGGAGCC CTAAACGCTCCGCGATCG
Axin2_Ab_BrP	CGG GAG ATT CGT CCT CAT AA AAC CGA GCG CGT AAC AGC AA GCG GTC TGT ATC CGA ATC TCA CGC TAT A AA ACC GCC GGC TTA GAA TAG AA CGA GGT CTT TCT AGC GGC

**SNAIL_Ab**	

SNAIL_Ab_tag	AAA AAA AAA ATAA CGC CGT ATC GCG ACT GCC GGA TTC GGT CGT AAA
SNAIL_Ab_PLP	AGTCGCGATACGGCGTTAATG CAT ATG CTA CAG GCT GGG GTG GAA CTGCGTCTATTTAGTGGAGC CTTTACGACCGAATCCGGC
SNAIL_Ab_BrP	GGA TCC AAG CGC TCG TAA AA GCG GCA TTG CGT TCA ACC AA ATG CAT ATG CTA CAG GCT GGG GTG GAA CAA ACC GCC GGC TTA GAA TAG AA CGA GGT CTT TCT AGC GGC

**N-cadherin_Ab**	

N-cadherin_Ab_tag	AAA AAA AAA AGTT GTC GGA TCG AGA GGC GAC CGT CGT TAG GTA TGC
N-cadherin_Ab_PLP	GCCTCTCGATCCGACAACAAC TTA CGC CCA AAG ACG GTA ACT TAG CTGCGTCTATTTAGTGGAGCC GCATACCTAACGACGGTC
N-cadherin_Ab_BrP	TAG GTA CGG GGC GCT TAA AA AAC CGA GCG CGT AAC AGC AA AAC TTA CGC CCA AAG ACG GTA ACT TAG CAA ACA TCG GGT ATG CGA GCA AA CGA GGT CTT TCT AGC GGC

**CDX2_Ab**	

CDX2_Ab_tag	AAA AAA AAA AACA TTG CGG CGT AAC ACC GGC TCG CAC GAC GTA TAC
CDX2_Ab_PLP	GGTGTTACGCCGCAATGTAAG GAA CCT AAG ATT GCG TGC GCC TTA ATGCGTCTATTTAGTGGAGCCG TATACGTCGTGCGAGCC
CDX2_Ab_BrP	GGA TCC AAG CGC TCG TAA AA GCC GTC AGT TCG GTT AAG AA AAG GAA CCT AAG ATT GCG TGC GCC TTA AAA CCACGGTCGGTCAATTCC AA CGA GGT CTT TCT AGC GGC

**FOXA2_Ab**	

FOXA2_Ab_tag	AAA AAA AAA ATCG ACT AAC TTG ACC TCA ATG AGA TGC ACG GGC CTC
FOXA2_Ab_PLP	TGAGGTCAAGTTAGTCGACCA TGC ATC GTC GTC CAA ATA TGT GAT CTGCGTCTATTTAGTGGAGC CGAGGCCCGTGCATCTCAT
FOXA2_Ab_BrP	GGA TCC AAG CGC TCG TAA AA GCC GTC AGT TCG GTT AAG AACCA TGC ATC GTC GTC CAA ATA TGT GAT CAA AGT ACG ATC GTG TAG CGG AA CGA GGT CTT TCT AGC GGC

**Sox2_Ab**	

Sox2_Ab_tag	AAA AAA AAA A GAA GCG CGG ATC ATC CTT CAC CAC TCG TCT TAA GGA
Sox2_Ab_PLP	AAGGATGATCCGCGCTTCGTA ATA CGC GTC AGA CCG CAC GCG GAC TTGCGTCTATTTAGTGGAG CCTCCTTAAGACGAGTGGTG
Sox2_Ab_BrP	TAG GTA CGG GGC GCT TAA AA GCG GCA TTG CGT TCA ACC AA GTA ATA CGC GTC AGA CCG CAC GCG GAC TAA CCA CGG TCG GTC AAT TCC AA CGA GGT CTT TCT AGC GGC

**TCF1_RNA**	

TCF1_Ex_PLP	CCACCACAGAGAGGTTTTTAA CGG TGT AGC TGT GGG CCA AGT CTA CTGCGTCTATTTAGTGGAGCC TGTGGCAAGGAACCATGT
TCF1_Ex_BrP	TAG GTA CGG GGC GCT TAA AA GCC GTC AGT TCG GTT AAG AA TAA CGG TGT AGC TGT GGG CCA AGT CTA C AA ACC GCC GGC TTA GAA TAG AA CGA GGT CTT TCT AGC GGC

**TCF3_RNA**	

TCF3_Ex_PLP	AGAACCGTCCTGATGCACCCA GTG TGT CAA ATC TCC GCA TTG GTA CTGCGTCTATTTAGTGGAGCC TGGAGACCTGTCTCATCC
TCF3_Ex_BrP	GCT ATG GTC TCT CGC TCC AA CGC CCG ATA AAC GGT ACA AA CCA GTG TGT CAA ATC TCC GCA TTG GTA C AA CCA CGG TCG GTC AAT TCC AA CGA GGT CTT TCT AGC GGC

**RB_RNA**	

RB_Ex_PLP	GTTGTGGCCAAACTTGACGCC ACT GGT AAT GCC ATT GGT CTA AAT GTGCGTCTATTTAGTGGAGCC AGGGTCCTCTTAAGCACA
RB_Ex_BrP	GGA TCC AAG CGC TCG TAA AA GCC GTC AGT TCG GTT AAG AA GCC ACT GGT AAT GCC ATT GGT CTA AAT G AA ACA TCG GGT ATG CGA GCA AA CGA GGT CTT TCT AGC GGC

**p21_RNA**	

p21_Ex_PLP	CCAGACCAGGATGTTACAGTC GTC TTG GTC GTA ACG AAT CTC CGT CTGCGTCTATTTAGTGGAGCC GGGCTAAGGGTAGACAGT
p21_Ex_BrP	TAG GTA CGG GGC GCT TAA AA CGC CCG ATA AAC GGT ACA AA GTC GTC TTG GTC GTA ACG AAT CTC CGT C AA CCA CGG TCG GTC AAT TCC AA CGA GGT CTT TCT AGC GGC

**p53_RNA**	

p53_Ex_PLP	CCAGCAGAGACCTGACAAGCT TCA GGC ATG ACA GGG TTG TCC ATG ATGCGTCTATTTAGTGGAGC CGGATAGAATTTCGCTGGG
p53_Ex_BrP	GCT ATG GTC TCT CGC TCC AA CGC CCG ATA AAC GGT ACA AA GCT TCA GGC ATG ACA GGG TTG TCC ATG A AA AGT ACG ATC GTG TAG CGG AA CGA GGT CTT TCT AGC GGC

**CyclinD1_RNA**	

Ccnd1_Ex_PLP	CCTCTGGCATTTTGGAGACCG GGT GAA TCT TTC GGG TGT CCA TGT ATGCGTCTATTTAGTGGAG CCTCTGCTTGTTCTCATCCG
Ccnd1_Ex_BrP	GCT ATG GTC TCT CGC TCC AA CGC CCG ATA AAC GGT ACA AA CCG GGT GAA TCT TTC GGG TGT CCA TGT A AA ACC GCC GGC TTA GAA TAG AA CGA GGT CTT TCT AGC GGC

**CDK4_RNA**	

CDK4_Ex_PLP	GGATCTTACGCTCGGCTACCT TTG ATC TCC TCC ACG CTA TAA GGT CTGCGTCTATTTAGTGGAG CCAGCCATTCTCGAAGCAGG
CDK4_Ex_BrP	GCT ATG GTC TCT CGC TCC AA CGC CCG ATA AAC GGT ACA AA CCT TTG ATC TCC TCC ACG CTA TAA GGT C AA ACA TCG GGT ATG CGA GCA AA CGA GGT CTT TCT AGC GGC

**CDK2_RNA**	

CDK2_Ex_PLP	CTTCAGTCTCAGTGTCGAGGA TAC CTA TAT GTC GGC CTG AAA CTC GTGCGTCTATTTAGTGGAG CCTGGCAGTACTGGGTACAC
CDK2_Ex_BrP	GCT ATG GTC TCT CGC TCC AA GCG GCA TTG CGT TCA ACC AA GGA TAC CTA TAT GTC GGC CTG AAA CTC G AA CCA CGG TCG GTC AAT TCC AA GTT AAC CAC TGT CGG GAC

**CyclinE1_RNA**	

Ccne1_Ex_PLP	CCGGATAACCATGGCGAAACT TGG TTG GCC GAC AAG ACT ACA TCC TTGCGTCTATTTAGTGGA GCCCTTGGAACTTCCCATCTC
Ccne1_Ex_BrP	GCT ATG GTC TCT CGC TCC AA GCG GCA TTG CGT TCA ACC AA ACT TGG TTG GCC GAC AAG ACT ACA TCC T AA AGT ACG ATC GTG TAG CGG AA GTT AAC CAC TGT CGG GAC

**STAT3_RNA**	

STAT3_Ex_PLP	GGTCAATCTTGAGGCCTTCGT TCC TTA TTT CCC GGT TGA GAT ACC ATGCGTCTATTTAGTGGAGC CGCAAGGAGTGGGTCTCTA
STAT3_Ex_BrP	TAG GTA CGG GGC GCT TAA AA CGC CCG ATA AAC GGT ACA AA CGT TCC TTA TTT CCC GGT TGA GAT ACC A AA AGT ACG ATC GTG TAG CGG AA CGA GGT CTT TCT AGC GGC

**AkT_RNA**	

AkT_Ex_PLP	CCACATGTGTGGTCTCAAGAG GTA TCA TGC GAG GCC GAA TCA TGG ATGCGTCTATTTAGTGGAG CCTTGTTCTGGGCACTGAGG
AkT_Ex_BrP	TAG GTA CGG GGC GCT TAA AA CGC CCG ATA AAC GGT ACA AA GAG GTA TCA TGC GAG GCC GAA TCA TGG A AA ACC GCC GGC TTA GAA TAG AA CGA GGT CTT TCT AGC GGC

**Smad2_RNA**	

Smad2_Ex_PLP	ATACTTTGTCCAGCCACTAAT ACC CGT AGG ACC AGT ACC GTC TTC GTGCGTCTATTTAGTGGAGC CGGGATCCCATCTGAGTTA
Smad2_Ex_BrP	CGG GAG ATT CGT CCT CAT AA GCC GTC AGT TCG GTT AAG AA AAT ACC CGT AGG ACC AGT ACC GTC TTC G AA AGT ACG ATC GTG TAG CGG AA CGA GGT CTT TCT AGC GGC

**Beta-catenin_RNA**	

Ctnnb1_Ex_PLP	TGGCTTGTCCTCAGACATGAC GGT TAA TCG CCT GGC CCA CTA TTA ATGCGTCTATTTAGTGGA GCCCCGCTTCTTGTAATCCTG
Ctnnb1_Ex_BrP	CGG GAG ATT CGT CCT CAT AA GCC GTC AGT TCG GTT AAG AA GAC GGT TAA TCG CCT GGC CCA CTA TTA A AA ACC GCC GGC TTA GAA TAG AA CGA GGT CTT TCT AGC GGC

**E-cadherin_RNA**	

Cdh1_Ex_PLP	TAGCGGCTTCAGAACCACGAG CGG GCT CTC GAT CAT CAT TCA TTT ATGCGTCTATTTAGTGGA GCCAGTTCAGTGAGCTCAGGC
Cdh1_Ex_BrP	CGG GAG ATT CGT CCT CAT AA GCC GTC AGT TCG GTT AAG AA GAG CGG GCT CTC GAT CAT CAT TCA TTT A AA ACA TCG GGT ATG CGA GCA AA CGA GGT CTT TCT AGC GGC

**N-caherin_RNA**	

Cdh2_Ex_PLP	CCTCCATAGTCTATGCTGTCT TCC ACC GAG GAG TAC GCC TAC ATT GTGCGTCTATTTAGTGGA GCCTTCCGAGTTCTCTGCACT
Cdh2_Ex_BrP	GGA TCC AAG CGC TCG TAA AA CGC CCG ATA AAC GGT ACA AA TCT TCC ACC GAG GAG TAC GCC TAC ATT G AA CCA CGG TCG GTC AAT TCC AA CGA GGT CTT TCT AGC GGC

**YAP1_RNA**	

YAP1_Ex_PLP	CACATTTGTCCCAGGGAGCCT AAG TAT TCG ATG CCG AAG TGT CCT ATGCGTCTATTTAGTGGA GCCTTCCAGTGTGCCAAGGTC
YAP1_Ex_BrP	GGA TCC AAG CGC TCG TAA AA CGC CCG ATA AAC GGT ACA AA CCT AAG TAT TCG ATG CCG AAG TGT CCT A AA AGT ACG ATC GTG TAG CGG AA CGA GGT CTT TCT AGC GGC

**Brachyury_RNA**	

T_Ex_PLP	GAGCCTAGAAGATCCAGTTAC TAG GTG GTG CCC ACA CGC ATA AGA TTGCGTCTATTTAGTGG AGCCCCAAGAGCCTGCCACTTT
T_Ex_BrP	CGG GAG ATT CGT CCT CAT AA CGC CCG ATA AAC GGT ACA AA TAC TAG GTG GTG CCC ACA CGC ATA AGA T AA AGT ACG ATC GTG TAG CGG AA CGA GGT CTT TCT AGC GGC

**Sox17_RNA**	

Sox17_Ex_PLP	ACTTGCCTAGCATCTTGCGTA TAG GTC GGG CAG GTC AGT TAT GAC CTGCGTCTATTTAGTGG AGCCAGGTCAACGCCTTCCAAG
Sox17_Ex_BrP	GGA TCC AAG CGC TCG TAA AA CGC CCG ATA AAC GGT ACA AA GTA TAG GTC GGG CAG GTC AGT TAT GAC C AA ACC GCC GGC TTA GAA TAG AA CGA GGT CTT TCT AGC GGC

**GATA6_RNA**	

GATA6_Ex_PLP	GCACAGGTAATGACCGGTACG GGA GTT CGG ATC TGC GTT AGA GTC CTGCGTCTATTTAGTGG AGCCGTAGAGACCGCATGCATT
GATA6_Ex_BrP	TAG GTA CGG GGC GCT TAA AA CGC CCG ATA AAC GGT ACA AA ACG GGA GTT CGG ATC TGC GTT AGA GTC C AA ACA TCG GGT ATG CGA GCA AA CGA GGT CTT TCT AGC GGC

**Axin2_RNA**	

Axin2_Ex_PLP	AACTGCTCCGCAGGCAAACAC GGT TTT ATC ACC GTG GTT CCC CTG ATGCGTCTATTTAGTGGA GCCGTCCCAGATCTCCTCAAA
Axin2_Ex_BrP	TAG GTA CGG GGC GCT TAA AA GCG GCA TTG CGT TCA ACC AA CAC GGT TTT ATC ACC GTG GTT CCC CTG A AA CCA CGG TCG GTC AAT TCC AA GTT AAC CAC TGT CGG GAC

**SNAIL1_RNA**	

SNAIL1_Ex_PLP	GGCATGGTTACAGCTGGACAG GAA CTA CAT GGC TGG CAT GGA TGC ATGCGTCTATTTAGTG GAGCCCAGTAACCACCCTGCTGA
SNAIL1_Ex_BrP	CGG GAG ATT CGT CCT CAT AA CGC CCG ATA AAC GGT ACA AA CAG GAA CTA CAT GGC TGG CAT GGA TGC A AA ACC GCC GGC TTA GAA TAG AA CGA GGT CTT TCT AGC GGC

**FOXA2_RNA**	

FOXA2_Ex_PLP	CTCTCATTTCCCTTGTCCACT AAC TGC ATG GGT GAT ACC GTT CTC CTGCGTCTATTTAGTGGA GCCCCAAAGTCTCCACTCAGC
FOXA2_Ex_BrP	TAG GTA CGG GGC GCT TAA AA GCG GCA TTG CGT TCA ACC AA ACT AAC TGC ATG GGT GAT ACC GTT CTC C AA AGT ACG ATC GTG TAG CGG AA GTT AAC CAC TGT CGG GAC

**Klf4_RNA**	

klf4_Ex_PLP	AACCACGACTCACCAAGCGAG CGT GAG GTT CCA TGG GAA AAT TAC GTGCGTCTATTTAGTGG AGCCCCCGTTTGGTACCTTTAG
klf4_Ex_BrP	GGA TCC AAG CGC TCG TAA AA GCG GCA TTG CGT TCA ACC AA GAG CGT GAG GTT CCA TGG GAA AAT TAC G AA CCA CGG TCG GTC AAT TCC AA GTT AAC CAC TGT CGG GAC

**Nanog_RNA**	

Nanog_Ex_PLP	AGCAGCATTCCAAGGCTGTCC TGG GCA ACT GGT ATC GGG CAG ATG GTGCGTCTATTTAGTG GAGCCCCCGAAGTTATGGAGCGG
Nanog_Ex_BrP	CGG GAG ATT CGT CCT CAT AA CGC CCG ATA AAC GGT ACA AA TCC TGG GCA ACT GGT ATC GGG CAG ATG G AA ACA TCG GGT ATG CGA GCA AA CGA GGT CTT TCT AGC GGC

**Smad1_RNA**	

Smad1_Ex_PLP	TTGTCTGTTTAAGGCAGCCAT TTA TTC CGC ACA ATC GCT CGA GTG CTGCGTCTATTTAGTGGAGC CGCAAATTCAGACACCAGC
Smad1_Ex_BrP	CGG GAG ATT CGT CCT CAT AA CGC CCG ATA AAC GGT ACA AA CAT TTA TTC CGC ACA ATC GCT CGA GTG C AA CCA CGG TCG GTC AAT TCC AA CGA GGT CTT TCT AGC GGC

**SRSF3_RNA**	

SRSF3_Ex_PLP	CAGTGGCTAAGACAACTAGAT GAG GCA AGT CTC CTA CAG CGC GGT GTGCGTCTATTTAGTGGAG CCTAAGACAAGGCACTAAGC
SRSF3_Ex_BrP	GGA TCC AAG CGC TCG TAA AA GCG GCA TTG CGT TCA ACC AA GAT GAG GCA AGT CTC CTA CAG CGC GGT GAA CCA CGG TCG GTC AAT TCC AA CGA GGT CTT TCT AGC GGC

**Sox2_RNA**	

Sox2_Ex_PLP	TGAAAATCTCTCCCCTTCTCCGA CCC TAG AAC GTT GGT GAT TGG CAT GTGCGTCTATTTAGTGGAG CCCCAATTCCCTTGTATCTCTT
Sox2_Ex_BrP	TAG GTA CGG GGC GCT TAA AA AAC CGA GCG CGT AAC AGC AA CGA CCC TAG AAC GTT GGT GAT TGG CAT GAA AGT ACG ATC GTG TAG CGG AA CGA GGT CTT TCT AGC GGC

**Oct4_RNA**	

Oct4_Ex_PLP	GGTTCTCTTGTCTACCTCAGG AAT AGG TCT CAC TGC TAT CGG CAC TTGCGTCTATTTAGTGGAGCCT TTAACCCCAAAGCTCCA
Oct4_Ex_BrP	TAG GTA CGG GGC GCT TAA AA GCC GTC AGT TCG GTT AAG AA AGG AAT AGG TCT CAC TGC TAT CGG CAC T AA ACA TCG GGT ATG CGA GCA AA CGA GGT CTT TCT AGC GGC

**P16Ink4a_RNA**	

P16Ink4a_PLP	CATCACCTGAATCGGGGTACG TTA GTT ATG TGG GTG GCT GAG CAA CTGCGTCTATTTAGTGGAG CCGTGAACGTTGCCCATCAT
P16Ink4a_BrP	GCT ATG GTC TCT CGC TCC AA GCG GCA TTG CGT TCA ACC AA ACG TTA GTT ATG TGG GTG GCT GAG CAA C AA ACC GCC GGC TTA GAA TAG AA GTT AAC CAC TGT CGG GAC

**p19Arf_RNA**	

p19Arf_PLP	CATCATCACCTGGTCCAGAAG TGT ACC AGG CAC TGA TTC GTT ACC GTGCGTCTATTTAGTGGAG CCTACGTGAACGTTGCCCAT
p19Arf_BrP	GCT ATG GTC TCT CGC TCC AA GCG GCA TTG CGT TCA ACC AA AAG TGT ACC AGG CAC TGA TTC GTT ACC G AA ACA TCG GGT ATG CGA GCA AA GTT AAC CAC TGT CGG GAC

The probe set for 67 targets with their sequences.

With five rounds of sequential hybridization, we measured 67 targets including 30 mRNAs and 37 (phospho-)proteins (Table 3). In the fifth round, we used two channels to repeat the targets detected in the first round. This allowed us to determine the reproducibility of the sequential hybridization. The other 4 rounds were included as a proof of principle, to show that multiple rounds were possible to allow for targeting more than 67 targets. Notably, the number of hybridization rounds can be amended according to the needs of the experiment. For other applications of this technique, we recommend integrating high-abundance and low-abundance targets within a single channel to prevent optical crowding.

This part provides the instructions to designing specific probes.

1. The 5′- and 3′- arm sequence of antibody-tagging ssDNA and antibody PLPs:
  a. Obtain the orthogonal DNA sequences from previous published dataset^8^ to generate a set of 36 nt probes for each protein target.
    ***Note:*** These sequences can be downloaded from Database: https://elledgelab.med.harvard.edu/?page_id=638. Each probe should have the first 18 nt corresponding to the 5′ arm and the last 18 nt corresponding to the 3′ arm of the antibody-tagging ssDNA (Figure 1A).
  b. Use the online OligoAnalyzer Tool (Integrated DNA technologies, https://eu.idtdna.com/pages/tools/oligoanalyzer) to get the reverse complementary sequence (See the screenshot below).
    ***Note:*** The first 18 nt sequences of the complementary sequence is the 5′- and the other 18 nt sequence is the 3′- arm of the antibody PLPs.

**Figure:**
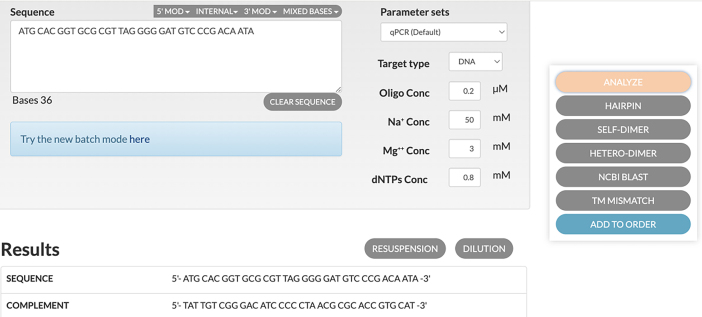
  c. Use NCBI Nucleotide BLAST (https://blast.ncbi.nlm.nih.gov/Blast.cgi) to identify and eliminate sequences with contiguous homology regions more than 70% to the mouse transcriptome. See the screenshot below:

**Figure:**
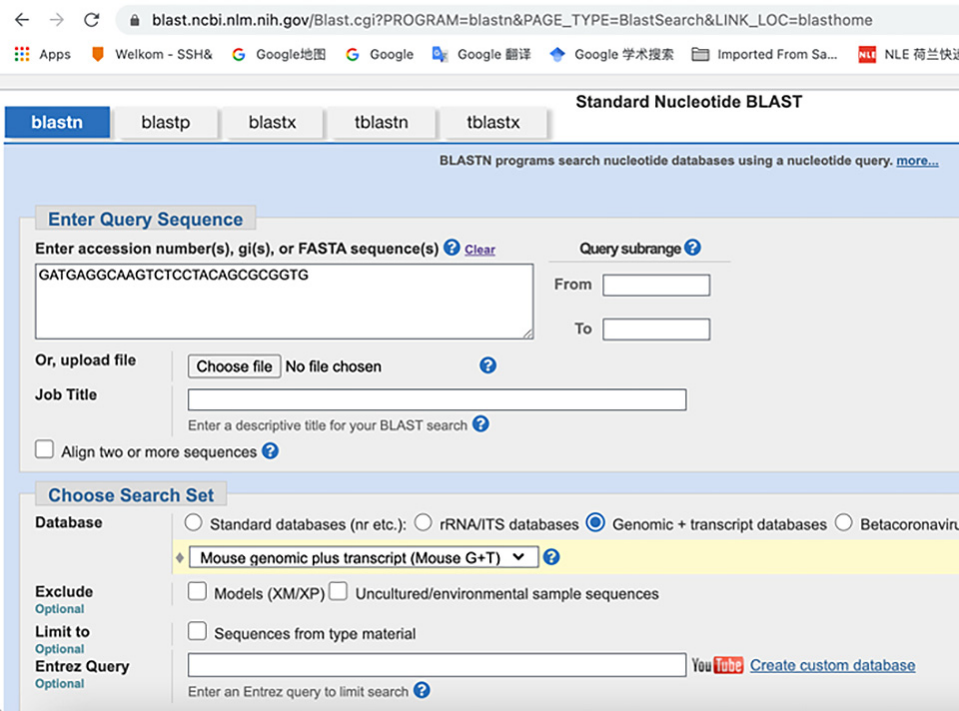

**Figure:**
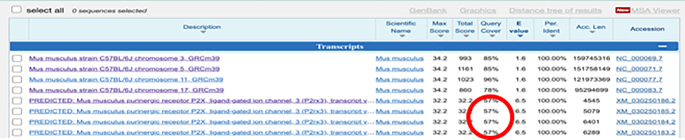

    **CRITICAL:** The 5′ end of antibody-tagging ssDNAs needs to be modified with azide to facilitate the conjugation reaction.
    **CRITICAL:** The 5′ end of PLP needs to be phosphorylated to facilitate the following ligation reactions.
2. The 5′- and 3′- arm sequence of mRNA PLPs (Figure 1B):
  a. Obtain the mRNA sequences of interest from NCBI website (*Mus musculus*). Refer to the example of the *Nanog* mRNA sequence obtained from NCBI in the screenshot below:

**Figure:**
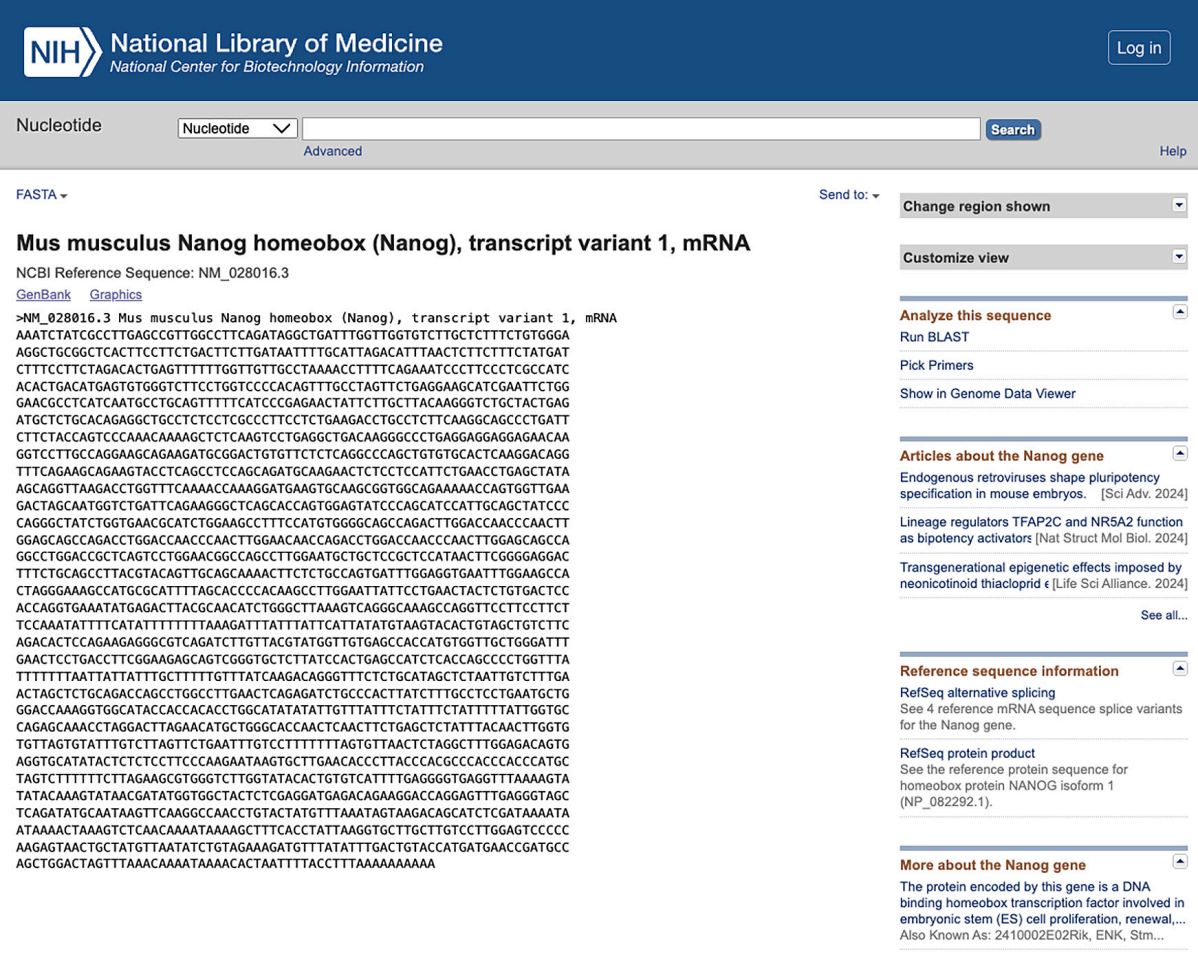
  b. Utilize the Stellaris Probe Designer from Biosearch Technologies (https: //www.biosearchtech.com/support/tools/design-software/stellaris-probe-designer) to generate a set of 36 nt probes that are complementary to the target mRNA sequence.
    ***Note:*** As shown in the screenshot below, set up the requirements for the probes. For example, type the probe name (i.e., Nanog_mRNA), choose the organism (i.e., Mouse), oligo length (i.e., 18 nt), and the Min. spacing length (i.e. 0 nt).

**Figure:**
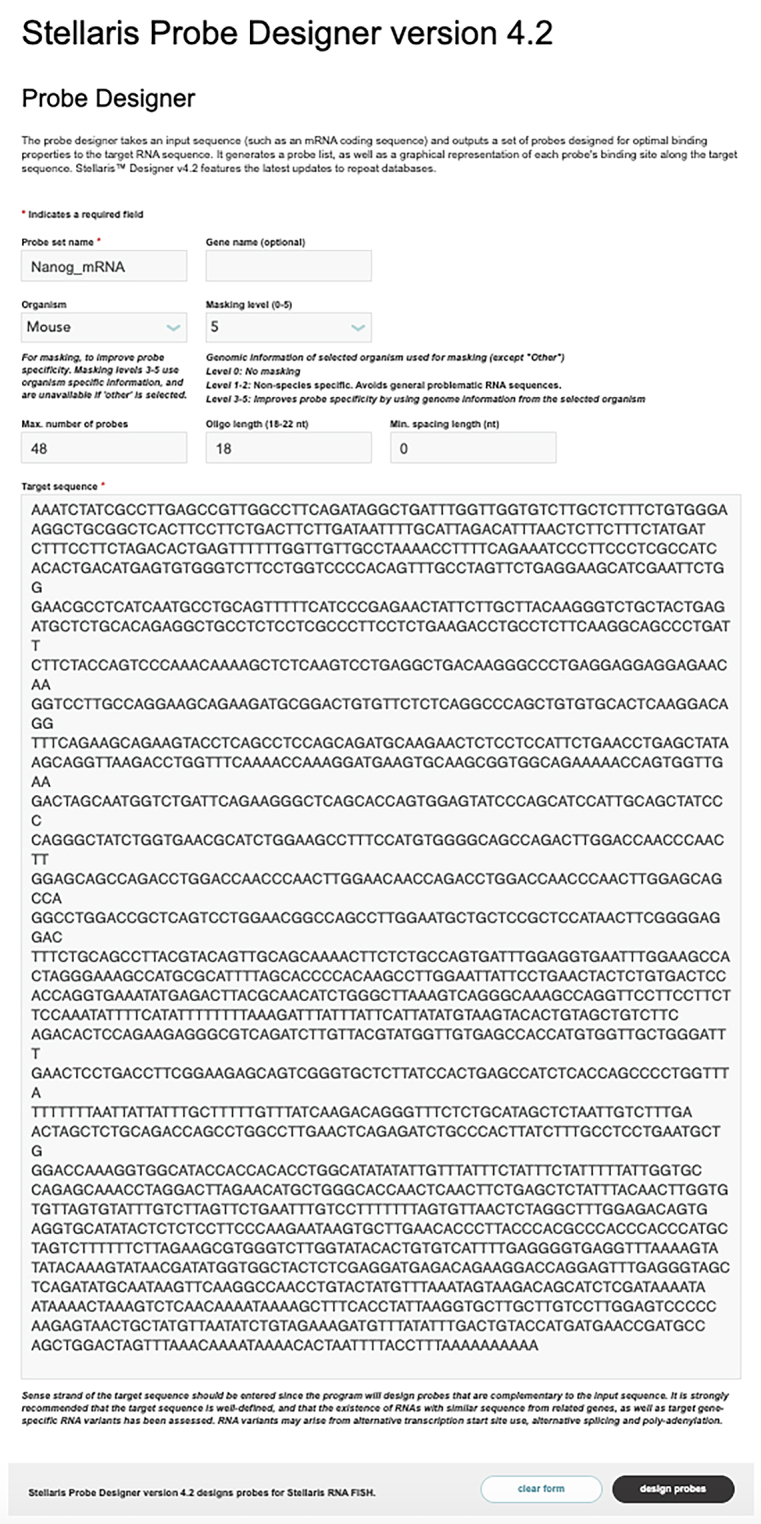
    Set the minimum spacing to 0 nt, as we aim to select a 36 nt probe from two neighboring 18 nt probes, such as Probe #1 and Probe #2, or Probe #3 and Probe #4 in the screenshot below. Each probe has the first 18 nt corresponding to the 5′ arm and the last 18 nt corresponding to the 3′ arm of the mRNA padlock probes (Figure 1B).

**Figure:**
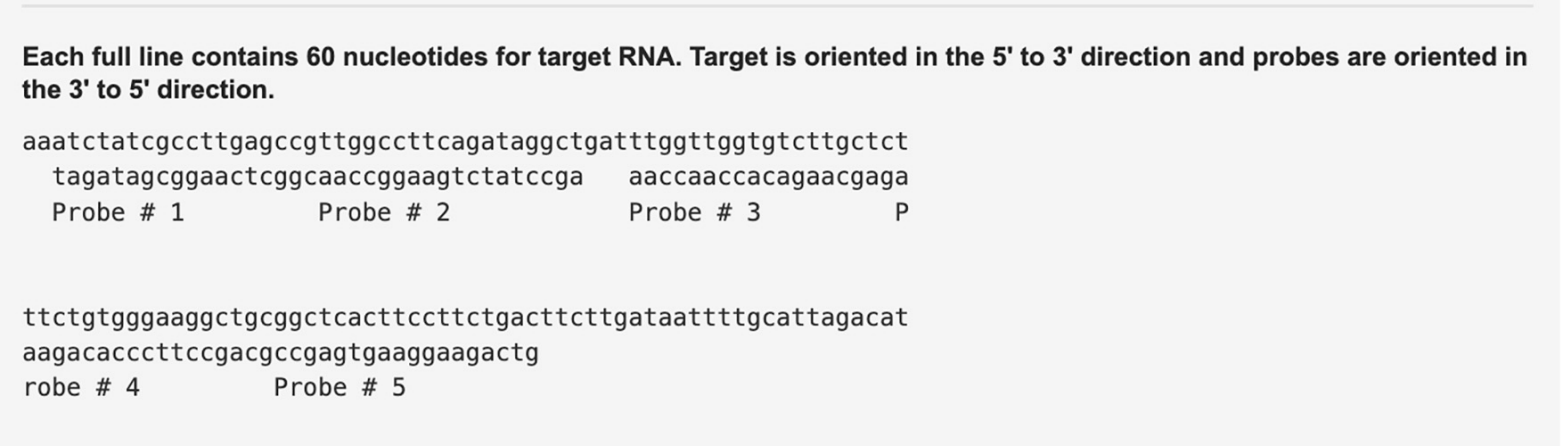
  c. Use NCBI Nucleotide BLAST (https://blast.ncbi.nlm.nih.gov/Blast.cgi) to ensure that the selected target sequence is unique and not present in other mRNAs within the same organism.

**Figure:**
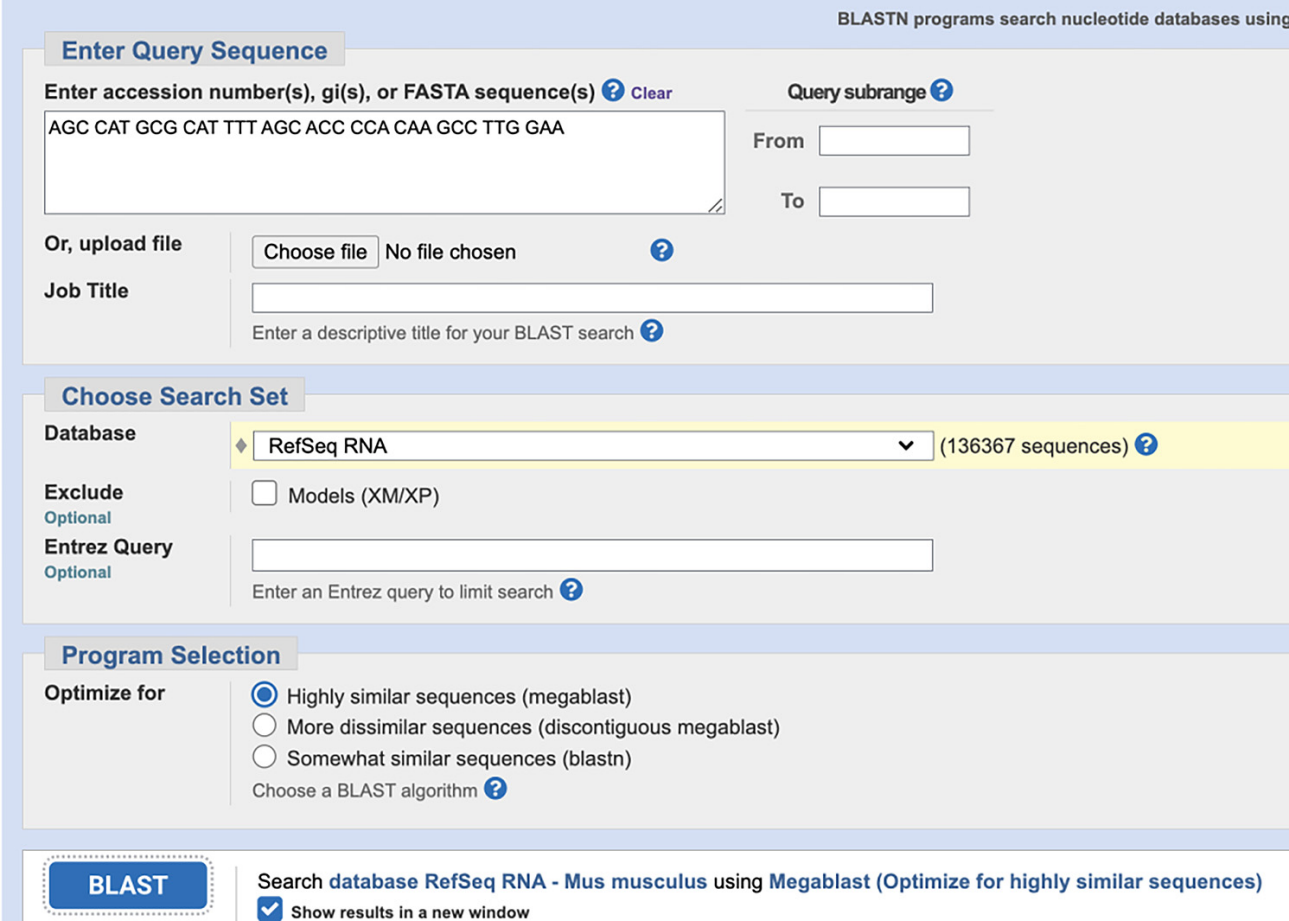

**Figure:**
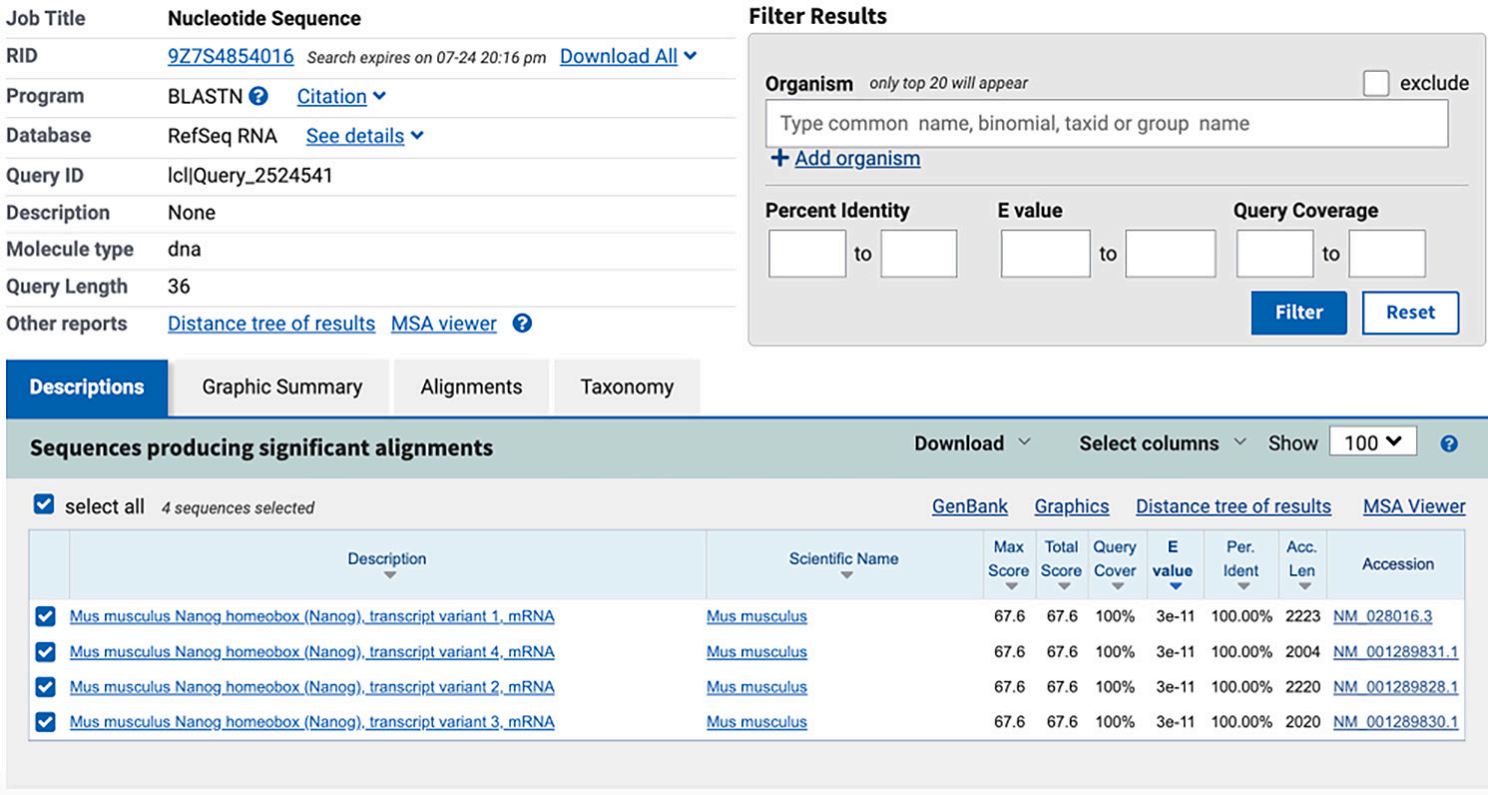

    **CRITICAL:** The 5′ end of PLP needs to be phosphorylated to facilitate the following ligation reactions.
    ***Note:*** Alternatively, write custom scripts or employ other tools for mRNA probe design.
3. The remaining sequences on the PLPs:
  a. Download the orthogonal DNA sequences from previous published dataset (Database: https://elledgelab.med.harvard.edu/?page_id=638)^8^ for the 28 nt ID sequences of each target (Figure 1B).
  b. Obtain a 20 nt orthogonal DNA sequence from previous published dataset (Database: https://elledgelab.med.harvard.edu/?page_id=638) for the RCA primer binding region on the PLPs (Figure 1B).
    ***Note:*** Both RNA and protein targets share the same RCA primers.
  c. Use NCBI Nucleotide BLAST (https://blast.ncbi.nlm.nih.gov/Blast.cgi) to identify and eliminate sequences with contiguous homology regions more than 70% (see screen shots below: Query cover) to the mouse genomic and transcript databases.

**Figure:**
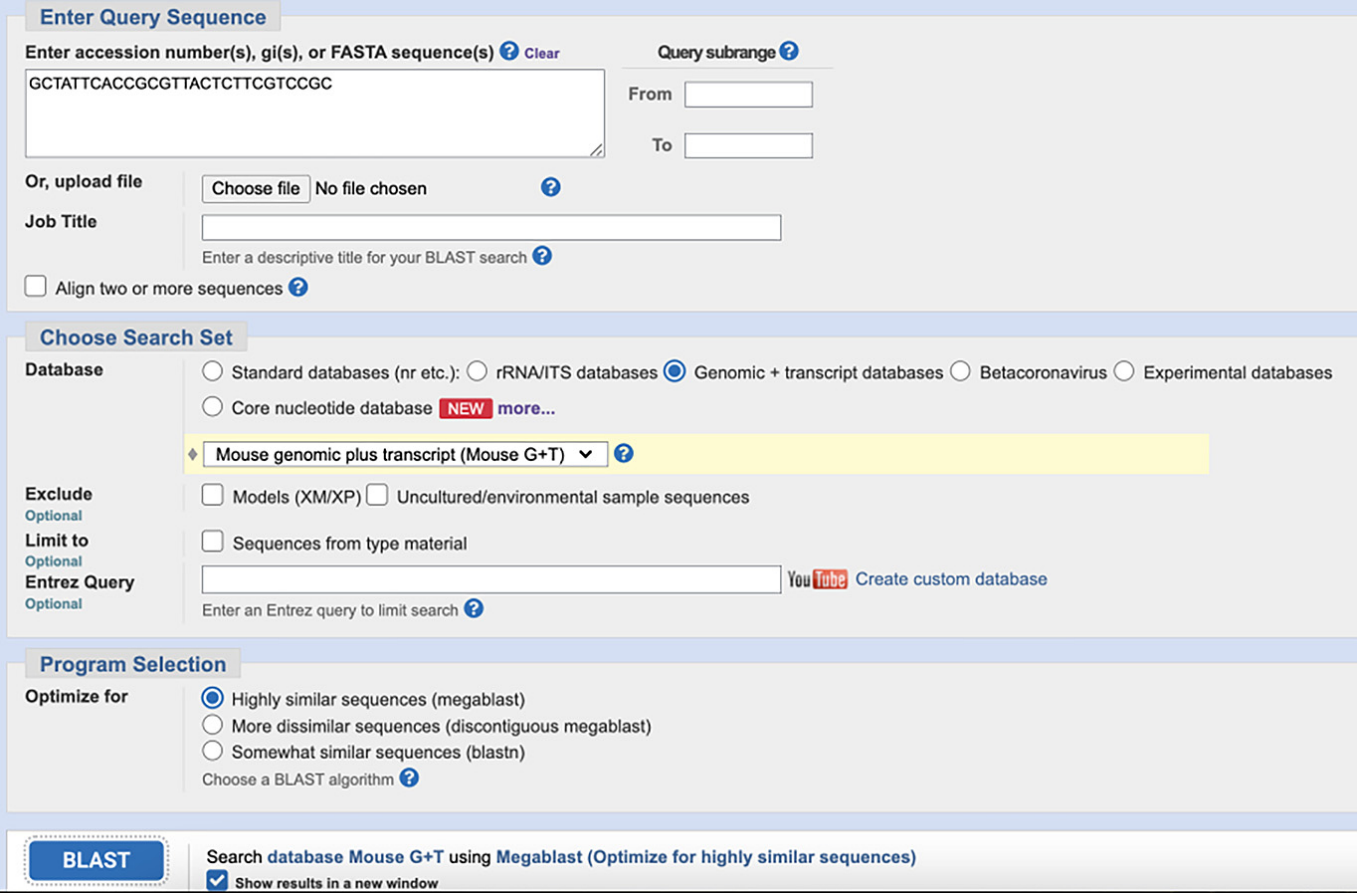

**Figure:**
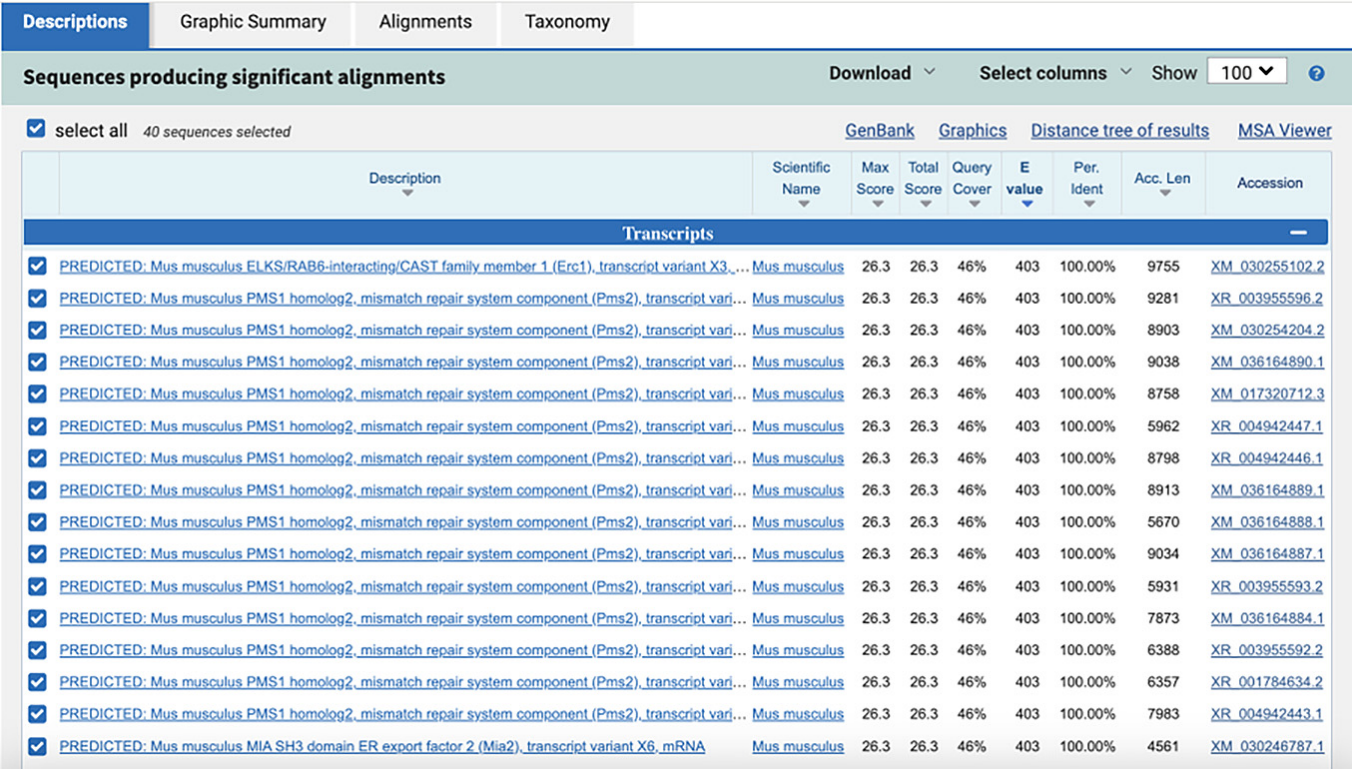
4. BrPs and RoPs sequences:
  a. Obtain 13 different 18 nt sequences from previously published orthogonal DNA sequences (Database: https://elledgelab.med.harvard.edu/?page_id=638)^8^ as RoPs. RoPs are conjugated with fluorophores, i.e., ATTO488, TAMRA, and CY5 (Figure 1D; Table 1).
  b. The four-overhang regions of the 18 nt sequences on BrPs are complementary to the four RoPs of each target. Use the online tools: OligoAnalyzer Tool (Integrated DNA technologies, https://eu.idtdna.com/pages/tools/oligoanalyzer) to get the reverse complementary sequences of RoPs (Figure 1C).
  c. Use BLAST to identify and eliminate sequences with contiguous homology regions more than 72% (see screen shots below: Query cover) to the mouse genomic and transcript databases.

**Figure:**
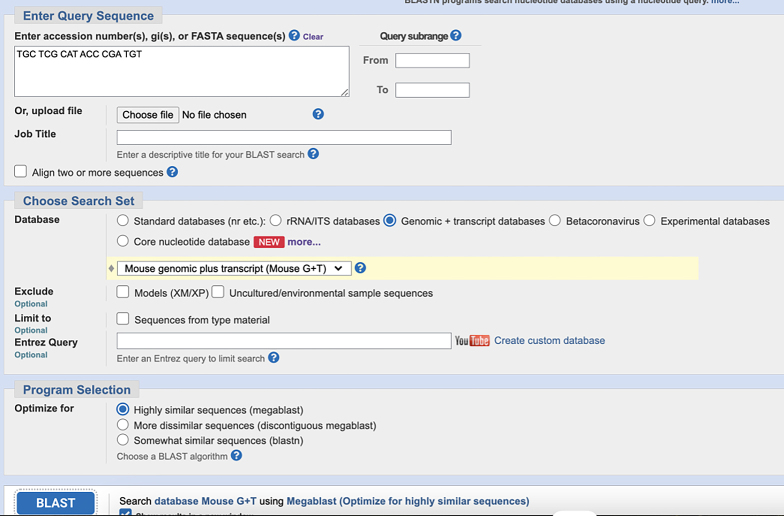

**Figure:**
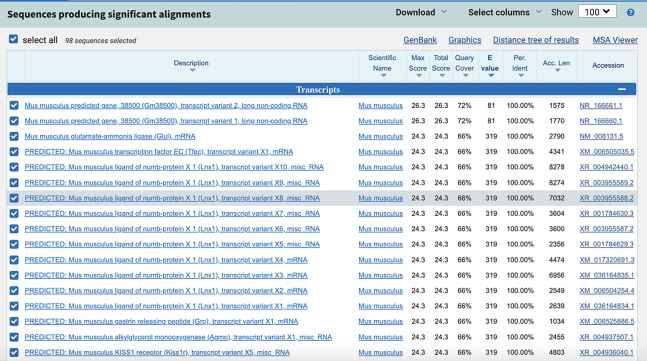

5. Purchase all the probes with the GC content within 45%–65% from an available supplier (e.g., Integrated DNA technologies (IDT)). The sequences of the probe are listed in Table 2.
6. Resuspend probes in Nuclease-free H_2_O (or TE buffer: 10 mM Tris pH 8.0, 0.1 mM EDTA) to a stock concentration of 10 μM. Store the probes at −20°C for a few years.
7. Aliquot out in multiple Eppendorf tubes and protect the fluorophore-labeled probes from the light by aluminum foil.

**CRITICAL:** Avoiding repeated freeze-thaw cycles to maintain the fluorescence stability of RoPs.

### Antibody conjugation

**Timing: 2 days**

This section outlines the protocol to conjugate 5′-azide modified ssDNA to an antibody. Each antibody is paired with its specific target ssDNA.

8. Prepare buffers - Day 1.
  a. 0.2 M NaHCO_3_ solution: dissolve 0.336 g NaHCO_3_ in 20 mL MilliQ water and adjust the PH to 8–9. Prepare this solution freshly every time.
  b. 1 mM DBCO-s-s-NHS: add 1.7 mL dimethylsulfoxide (DMSO) to 1 mg DBCO-s-s-NHS and mix well. Aliquot small volume (usage for once, for example: 10–50 μL per tube) into Eppendorf tubes Prepare this solution freshly every time.

***Note:*** DBCO-s-s-NHS is moisture-sensitive. To avoid moisture condensation onto the product always let the vial equilibrate to room temperature (i.e., around 22°C–25°C) before opening; be careful to limit exposure to moisture and restore under an inert atmosphere.

9. Buffer exchange for the antibodies in the form of solution - Day 1.
  a. Take out the antibodies (from their storage temperature, e.g., 4°C or –20°C) and equilibrate them to room temperature (i.e., around 22°C–25°C).
    ***Note:*** Antibodies are purchased from supplier with the requirements of being in lyophilized form or in PBS solution containing azide. The antibodies in a solution with bovine serum albumin (BSA) or glycerol cannot be used in this conjugation protocol.
  b. Calibration of the ZebaSpin column.
    i. Take ZebaSpin out from refrigerator.
    ii. Remove the bottom plastic part to allow liquid to pass through the solid phase during centrifugation and put the ZebaSpin in an Eppendorf tube.
    iii. Loosen the cap of the tube.
    iv. Centrifuge the ZebaSpin 40k (kilodaltons, kDa) column centrifuge unit for 1 min at 1500 rcf (relative centrifugal force).
    v. Wash 1: Discard the liquid in the Eppendorf tube and put the column back to the Eppendorf tube. Add 300 μL NaHCO_3_ buffer to the column. Centrifuge for 1 min at 1500 rcf.
    vi. Wash 2: Discard the liquid in the Eppendorf tube and put the column back to the Eppendorf tube. Add 300 μL NaHCO_3_ buffer to the column. Centrifuge for 1 min at 1500 rcf.
    vii. Wash 3: Discard the liquid in the Eppendorf tube and put the column back to the Eppendorf tube. Add 300 μL NaHCO_3_ buffer to the column. Centrifuge for 2 min at 1500 rcf.
    viii. Perform paper blotting: take Kimtech tissue and gently remove liquid from the tip of column.
    ix. Put ZebaSpin in a new Low-bind protein tube to collect antibody into the Low-bind Eppendorf.
  c. Buffer exchange:
    i. Add 100 μL antibody (50 μg antibody in total, in this case, the concentration of the antibody is 0.5 μg/μL) to the central part of the column.
      ***Note:*** The volume of the antibody dilution is not as relevant as its concentration. For this reason, it is recommended to utilize antibodies with starting concentrations between 0.5 μg/μL to 1 μg/μL.
    ii. Centrifuge for 2 min at 1500 rcf.
      **CRITICAL:** For lyophilized antibodies, buffer exchange is not required, and they can be directly dissolved in the buffer.
10. Functionalization of antibodies - Day 1.
  a. Add 10× molar excess of DBCO-s-s-NHS to 50 μg antibody.
    i. Add 3.3 μL DBCO-s-s-NHS (1 mM in DMSO) to 50 μg antibody (333.5 pmol antibody in 100 μL NaHCO_3_ pH > 8).
  b. Leave the mixed solution at room temperature (i.e., around 22°C–25°C) for at least 1 h in darkness.

**CRITICAL:** This is a light sensitive reaction.

11. Buffer exchange for the functionalized antibodies and removal of excessed DBCO-s-s-NHS - Day 1.
  a. Take out ZebaSpin 40k column from the refrigerator.
  b. Calibration of the ZebaSpin column.
    i. Remove the bottom plastic part to allow liquid to pass through the solid phase during centrifuge and put the ZebaSpin in an Eppendorf tube.
    ii. Loosen the cap of the tube.
    iii. Centrifuge the ZebaSpin 40k column centrifuge unit for 1 min at 1500 rcf.
    iv. Wash 1: Discard the liquid in the Eppendorf tube and put the column back to the Eppendorf tube. Add 300 μL NaHCO_3_ buffer to the column. Centrifuge for 1 min at 1500 rcf.
    v. Wash 2: Discard the liquid in the Eppendorf tube and put the column back to the Eppendorf tube. Add 300 μL NaHCO_3_ buffer to the column. Centrifuge for 1 min at 1500 rcf.
    vi. Wash 3: Discard the liquid in the Eppendorf tube and put the column back to the Eppendorf tube. Add 300 μL NaHCO_3_ buffer to the column. Centrifuge for 2 min at 1500 rcf.
    vii. Perform paper blotting: take Kimtech tissue and gently remove liquid from the tip of column.
    viii. Put ZebaSpin in a new Low-bind protein tube to collect antibody into the Low-bind Eppendorf.
  c. Buffer exchange:
    i. Add all the functionalized antibody to the central part of the column.
    ii. Centrifuge for 2 min at 1500 rcf.
12. Conjugation of the antibodies to ssDNA - Day 1.
  a. Mix 50 μL target-specific ssDNA (100 μM) with 50 μg functionalized antibodies by pipetting more than 20 times.
    ***Note:*** The molar ratio of antibody and the ssDNA is 1:15.
  b. Incubate at room temperature (i.e., around 22°C–25°C) for 1 h first and then move them to 4°C for overnight (about 16 h).
13. Preparation of buffers - Day 2.
  a. 2× concentrated antibody (Ab) solution: 50 mL DPBS with 200 μL 2 mM Ethylenediaminetetraacetic acid (EDTA) and 500 μL 0.1% Sodium azide.

***Note:*** Prepare this freshly every time. 0.1% Sodium azide is stored at 4°C. Sodium azide is extremely toxic. Handle with appropriate PPE and dispose of it according to hazardous waste guidelines.

14. Quench the conjugation reaction -Day 2.
  a. Add 100 μL 2× concentrated Ab solution to the sample.
15. Cleanup the conjugated antibodies - Day 2.
  a. Pre-wet the filter by adding 400 μL Blocking buffer in a Amicon ultra 100k centrifuge unit.
  b. Centrifuge for 5 min at 14000 rcf.
  c. Remove the flow-through.
  d. Add 400 μL 2× concentrated Ab solution (if the sample volume is less than 400 μL, top up with antibody buffer)
  e. Centrifuge for 10 min at 14000 rcf.
  f. Wash 1: Discard the flow through and add 400 μL 2× concentrated Ab solution.
  g. Centrifuge for 10 min at 14.000 rcf.
  h. Wash 2: Discard the flow through and add 400 μL 2× concentrated Ab solution.
  i. Centrifuge for 10 min at 14.000 rcf.
  j. Place the column up-side-down in a new Low-bind protein Eppendorf tube.
  k. Centrifuge for 10 min at 1000 rcf.
  l. The conjugated antibody solution is collected in the Low-bind protein Eppendorf tube.

***Note:*** If the loss is around 50%, the final concentration of the antibody will be around 25 μg in 50 μL, i.e., 0.5 μg/μL.

***Note:*** To confirm the successful conjugation of the antibodies, perform polyacrylamide gel electrophoresis (PAGE). Successful conjugation leads to a slight increase in the molecular weight of the antibody heavy chain, which can be easily detected by a noticeable upward shift on a gel.

## Key resources table

**Table:** 

REAGENT or RESOURCE	SOURCE	IDENTIFIER
**Antibodies**		

GATA-6 (1:4,000)	R&D	Cat# MAB1700
BRACHYURY (1:4,000)	R&D	Cat# MAB20851-100
Phospho-STAT3 (S727) (1:4,000)	R&D	Cat# MAB4934
Phospho-AKT1 (T308) (1:4,000)	R&D	Cat# MAB7419
AXIN2 (1:4,000)	R&D	Cat# MAB6078
TCF3/E2A (1:4,000)	R&D	Cat# MAB7650
CDX2 (1:4,000)	Abcam	Cat# ab220799
Phospho-YAP1 (S127) (1:4,000)	Abcam	Cat# ab172374
CDK4 (1:4,000)	Sigma	Cat# SAB1403657-100UG
RB (2M15) (1:4,000)	Sigma	Cat# ZRB1014-4X25UL
STAT3 (1:4,000)	Sigma	Cat# ZRB1004-4X25UL
TCF7 (TCF1) (1:4,000)	Sigma	Cat# WH0006932M1-100UG
NANOG (1:4,000)	Sigma	Cat# ZRB1566-4X25UL
p16-INK4a (1:4,000)	Sigma	Cat# ZRB1437-4X25UL
YAP (1:4,000)	Sigma	Cat# MABS2029-100UG
SMAD1 (1:4,000)	Merck	Cat# 05-1459
AKT1 (1:4,000)	Thermo Fisher Scientific	Cat# CF504235
OCT4 (3H8L1.12) (1:4,000)	Thermo Fisher Scientific	Cat# 703927
CDK2c (1A6) (1:4,000)	Thermo Fisher Scientific	Cat# MA5-17052
FOXA2 (9H5L7) (1:4,000)	Thermo Fisher Scientific	Cat# 701698
SOX17 (6H42L1) (1:4,000)	Thermo Fisher Scientific	Cat# 703063
Phospho-RNA POL II CTD (Ser5) (4H8) (1:4,000)	Thermo Fisher Scientific	Cat# MA1-46093
β-catenin (CAT-5H10) (1:4,000)	Thermo Fisher Scientific	Cat# 13-8400
Phospho-β-catenin (Thr41, Ser45) (23H16L13) (1:4,000)	Thermo Fisher Scientific	Cat# 703638
SMAD2 (31H15L4) (1:4,000)	Thermo Fisher Scientific	Cat# 700048
Phospho-SMAD2 (Ser245, Ser250) (2H24L4) (1:4,000)	Thermo Fisher Scientific	Cat# 701723
Cyclin E (HE12)	Thermo Fisher Scientific	Cat# 32-1600
Phospho-RB (Ser807, Ser811) (13H27L9) (1:4,000)	Thermo Fisher Scientific	Cat# 702097
N-Cadherin (CD325) (8C11) (1:4,000)	Thermo Fisher Scientific	Cat# 14-3259-82
E-Cadherin (CD324) (DECMA-1) (1:4,000)	Thermo Fisher Scientific	Cat# 16-3249-85
P21 (GT1032) (1:4,000)	Thermo Fisher Scientific	Cat# MA5-31479
P53 (X77) (1:4,000)	Thermo Fisher Scientific	Cat# MA1-12549
Cyclin D1 (AM29) (1:4,000)	Thermo Fisher Scientific	Cat# 33-3500
SNAIL1 (20C8) (1:4,000)	Thermo Fisher Scientific	Cat# 14-9859-82
SRSF3 (1:4,000)	Thermo Fisher Scientific	Cat# 334200
SOX2 (1:4,000)	Invitrogen	Cat# MA531455
Phospho-SMAD1 (Ser463, 465) (1:4,000)	Invitrogen	Cat# 700047

**Biological samples**		

mESC-E14	van Mierlo et al.^9^	N/A

**Chemicals, peptides, and recombinant proteins**		

Dulbecco’s modified Eagle’s medium	Gibco	Cat# 41965039
Gelatin	Sigma-Aldrich	Cat # 48723-500G
Fetal bovine serum	Gibco	Cat# A3840002
L-glutamine	Gibco	Cat# A2916801
Sodium pyruvate	Gibco	Cat# 11360039
Penicillin-streptomycin	Gibco	Cat# 15140122
Beta-mercaptoethanol	Gibco	Cat# 31350010
Recombinant leukemia inhibitory factor (LIF)	Millipore	Cat# ESG1107
Trypsin ethylenediaminetetraacetic acid (EDTA)	Gibco	Cat# 25300062
37% formaldehyde solution	Sigma-Aldrich	Cat# 1040021000-1L
DAPI (4′,6-diamidino-2-phenylindole)	Thermo Scientific	Cat# 62247
NaHCO_3_	Thermo Scientific	Cat# 217125000
DBCO-S-S-NH (dibenzocyclooctyne-S-S-N-hydroxysuccinimidyl) ester	Sigma-Aldrich	Cat# 761532-1mg
Phosphate-buffered saline (PBS)	Gibco	Cat# 20012019
Sodium azide	Sigma-Aldrich	Cat# S8032-25G
Ethylenediaminetetraacetic acid (EDTA)	Lonza	Cat# 51201
Bovine serum albumin (BSA)	Sigma-Aldrich	Cat# A4503-50G
Ampligase ligase buffer	Lucigen	Cat# A1905B
KCl (potassium chloride)	Invitrogen	Cat# AM9640G
Formamide	Invitrogen	Cat# AM9342
Yeast tRNA	Invitrogen	Cat#AM7119
RiboLock RNase inhibitor	Thermo Scientific	Cat# EO0381
Nuclease-free water	Invitrogen	Cat# AM9937
SplintR ligase	NEB	Cat# M0375
ATP	Invitrogen	Cat# 18330019
Phi29 polymerase	NEB	Cat# M0269L
Glycerol	Sigma-Aldrich	Cat# G5516
Deoxynucleotide triphosphates (dNTP)	Thermo Scientific	Cat# R0192
T4 DNA ligase	Thermo Scientific	Cat# EL0014
Exonuclease I	Thermo Scientific	Cat# EN0581
20× SSC (saline sodium citrate), RNase-free	Thermo Scientific	Cat# AM9763
Methanol	Sigma-Aldrich	Cat# 179957
Glucose oxidase	Sigma-Aldrich	Cat# G2133-10KU
Catalase	Sigma-Aldrich	Cat# C3515-10MG
Trolox	Sigma-Aldrich	Cat# 648471
Dextran sulfate	Sigma-Aldrich	Cat# D8906-10G

**Experimental models: Cell lines**		

Mouse embryonic stem cells	Gift from Hendrik Marks^9^	mESC-E14

**Oligonucleotides**		

Probes are listed in Tables 1 and 2	–	–

**Software and algorithms**		

Stellaris Probe Designer	Biosearch Technologies	https://www.biosearchtech.com/support/tools/design-software/stellaris-probe-designer
ImageJ (Fiji)	–	–
FlowJo v10.8.0 software	BD	N/A

**Other**		

μ-Slide 15 well 3D (formerly μ-Slide Angiogenesis)	ibidi	Cat# 81501
Zeba spin desalting columns, 40 K MWCO 0.5 mL	Thermo Scientific	Cat# 87767
100 K Amicon centrifuge filters	Merck	Cat# UFC510096
Parafilm	Fisher Scientific	Cat# 1337416
Fluorescence-activated cell sorting FACSCalibur flow cytometer	BD	–
Spinning disk confocal	Olympus	–

## Materials and equipment

**Fixation buffer:** Dilute formaldehyde to a 4% final concentration in 1× phosphate-buffered saline (PBS). Aliquot 40 mL into each 50 mL tube and store at −20°C. Once thawed, keep it at 4°C.

**CRITICAL:** Handle formaldehyde safely by wearing appropriate personal protective equipment and working in well-ventilated areas, while minimizing exposure through dilution and proper storage. Adhere to regulatory guidelines and emergency procedures to ensure a safe working environment.

**Permeabilization solution:** Aliquot 40 mL into each 50 mL tube and store 100% methanol at −20°C.

**CRITICAL:** Exercise caution when handling methanol by wearing appropriate personal protective equipment, working in well-ventilated areas, and avoiding inhalation and skin contact. Store methanol in tightly sealed containers away from heat and ignition sources and follow proper disposal procedures to minimize environmental impact.

**Blocking buffer:** Dissolve 0.5 g BSA in 10 mL 1× PBS to make a 5% BSA solution stock. Aliquot 1 mL in Eppendorf tubes and store at −20°C. Avoid thawing and freezing cycles.

**Deionized formamide**: Dilute 100% formamide with nuclease-free H_2_O to achieve a final concentration of 20% for hybridization reactions as shown in the following steps.

***Note:*** Formamide should be stored according to manufacturer-recommended conditions to maintain a deionized state.

**CRITICAL:** When working with formamide, prioritize safety by utilizing appropriate personal protective equipment, ensuring adequate ventilation, and minimize direct contact with skin or inhalation of vapors. Store formamide in tightly sealed containers in a cool, well-ventilated area, and adhere to proper disposal guidelines to mitigate environmental risks.

**Wash buffer (2**× **SSC, saline sodium citrate)**: Dilute 20× SSC to 2× SSC with nuclease-free H_2_O.

## Step-by-step method details

### Fixation and permeabilization of cells

**Timing: 10 min + 10 min**

#### Day 1

This step details the fixation and permeabilization of cellular samples in preparation for subsequent antibody staining. The protocol offers three options for permeabilization, allowing for optional hybridization.

1. Fix samples with 4% formaldehyde at room temperature (i.e., around 22°C–25°C) for 10 min.
  a. Remove the cell culture medium from the samples.
    ***Note:*** Add wash step before fixation if the background is high.
  b. Add 4% formaldehyde (equivalent volume to cell culture medium) to the samples.
  c. Incubate in fixation solution for 10 min at room temperature (i.e., around 22°C–25°C).
  d. Remove fixation solution from the samples.
  e. Wash the samples three times with 1× PBS.
    ***Note:*** Mouse embryonic stem cells are used as samples in this study. For the optimization experiments, there are 5∗10ˆ4 cells seeded in each well of the μ-Slide 15 Well 3D (formerly μ-Slide Angiogenesis). Cells are fixed 24 h after seeding.
    ***Note:*** Samples that are co-cultured in the same slide or plate, even when in different wells, must be fixed in parallel (at the same time). Otherwise, the vapors from fixing one well will affect the outcomes of wells that remain unfixed.
2. Permeabilize samples with pre-chilled (at −20°C) 100% methanol for 10 min at −20°C.
  a. Remove 1× PBS solution from the samples, corresponding to last wash.
  b. Add pre-chilled 100% methanol (equivalent volume to cell culture medium) to each sample.
  c. Incubate in 100% methanol for 10 min at −20°C.
  d. Remove fixation solution from the samples.
  e. Wash the samples three times with 1× PBS.

**Figure 2:**
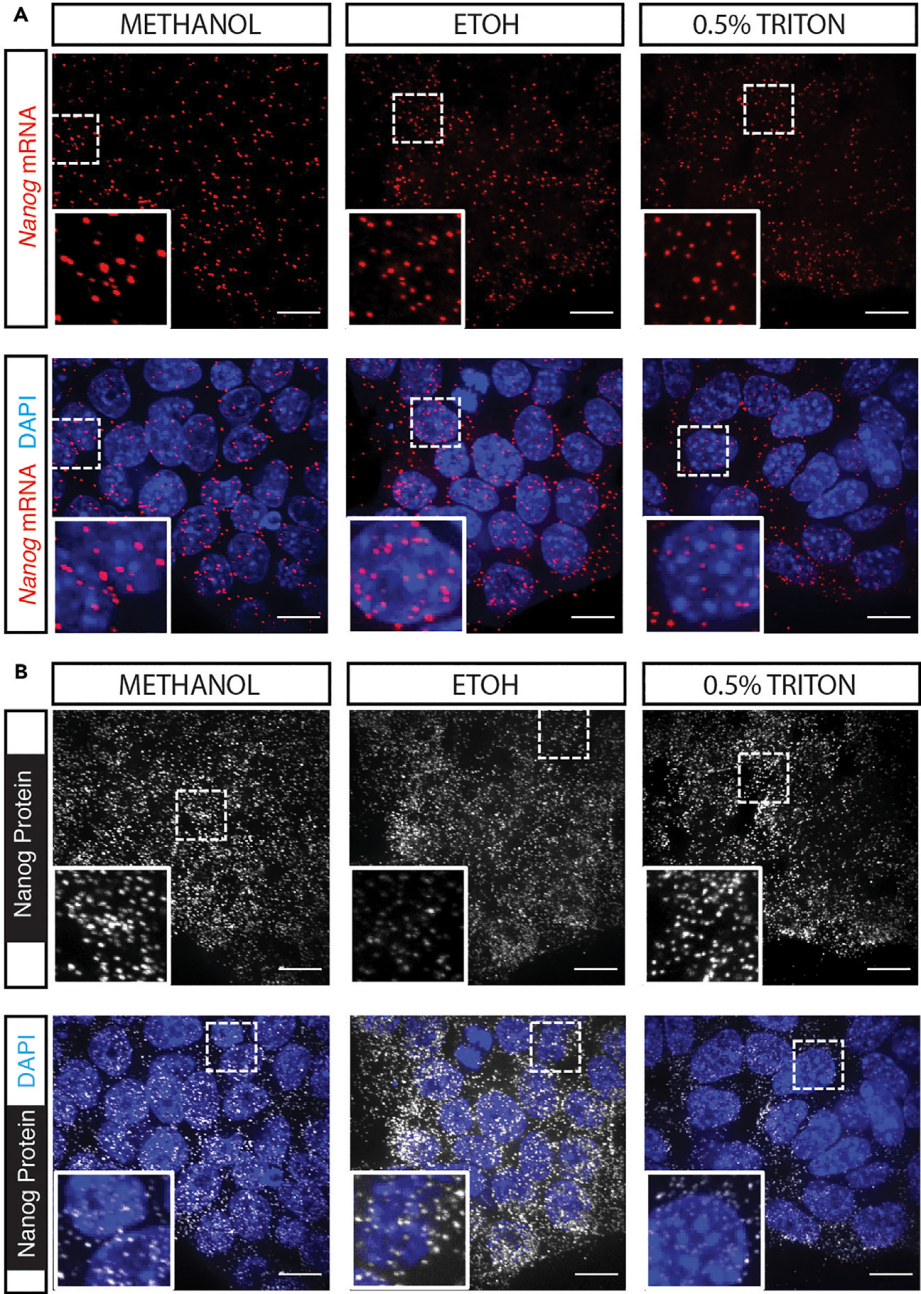
Different permeabilization methods Representative z-projection images showing mRNA (A), and protein (B) detected by ARTseq-FISH with methanol, 70% ethanol, or 0.5% Triton in PBS permeabilization. DAPI shows the nuclei staining. Scale bar, 20 μm.

***Alternatives:*** Alternative permeabilization strategies, such as using 70% ethanol or 0.1%–0.5% Triton X-100, can also be employed to simultaneously detect both protein and mRNA targets (Figure 2). However, the concentration of Triton X-100 may need optimization for optimal results.

### Blocking and primary antibodies incubation

**Timing: 5 h**

This step describes the blocking and antibody incubation procedures of ARTseq-FISH as a general immunofluorescence method. Blocking reduces the unspecific antibody binding.

3. Add blocking buffer (equivalent volume to cell culture medium) to samples.
4. Incubate for 1 h at room temperature (i.e., around 22°C–25°C).
5. Incubate with ssDNA-conjugated antibody.
  a. Prepare antibody solution: dilute ssDNA-conjugated antibodies with a ratio of 1:4000 in blocking buffer.
  b. Remove the blocking buffer from the sample.
  c. Add 30 μL antibody solution per well.
  d. Incubate for 4 h at room temperature (i.e., around 22°C–25°C).

**CRITICAL:** We have optimized the order of antibody incubation and the mRNA PLP hybridization. When combined, perform the antibody incubation first followed by the mRNA PLP hybridization for a better outcome.

### mRNA padlock probe (PLP) hybridization

**Timing: 16 h (overnight)**

This step provides the protocol of the hybridization of mRNA to the PLPs. This is an essential step for the detection efficiency. The concentration of PLPs and the incubation temperature are critical to this step.

6. Prepare the mRNA PLP hybridization solution as below:

**Table:** 

	Reagent	Stock	Final	1× (μL)
1	NF-H_2_O (Nuclease free water)	–	–	up to 30 μL
2	10× Ampligase buffer	10×	1×	3 μL
3	KCl (Potassium chloride)	1 M	50 mM	1.5 μL
4	Formamide	100%	20%	6 μL
5	PLPs	10 μM/target	100 nM/target	0.3 μL/target
6	BSA	20 μg/μL	0.2 μg/μL	0.3 μL
7	tRNA (Transfer ribonucleic acid)	10 μg/μL	0.2 μg/μL	0.6 μL
8	RiboLock RNase Inhibitor	40 U/μL	1 U/μL	0.75 μL
	Total			30 μL/well

7. Remove the antibody solution from the samples.
8. Wash the samples three times with 30 μL 1× PBS.
9. Add 30 μL of the mRNA PLP hybridization solution prepared in step 6 to each sample.
10. Wrap the samples with Parafilm to prevent evaporation and aluminum foil prevented from light.
11. Incubate at 37°C overnight (about 16 h).

### mRNA padlock probe (PLP) ligation

**Timing: 4 h**

#### Day 2

This step describes the ligation of mRNA PLPs, which is another challenging step in this technique. The ligase and ligase buffer, the incubation time and temperature are critical for the efficiency of this step.

12. Prepare the mRNA PLP ligation solution as below:

**Table:** 

	Reagent	Stock	Final	1× (μL)
1	NF-H_2_O	–	–	up to 30 μL
2	10× Ampligase buffer	10×	1×	3 μL
3	RiboLock RNase Inhibitor	40 U μL^–1^	1 U/ μL	0.75 μL
4	SplintR Ligase	25 U μL^–1^	0.5 U/ μL	0.6 μL
5	ATP (Adenosine triphosphate)	1 mM	10 μM	0.3
6	BSA	20 μg/μL	0.2 μg/μL	0.3
	Total			30 μL/well

13. Remove the mRNA PLP hybridization solution from the samples.
14. Wash the samples three times with 30 μL 2× SSC.
15. Add 30 μL of the solution prepared above in step 12 to the samples.
16. Wrap the samples with Parafilm to prevent evaporation and aluminum foil to protect from light.
17. Incubate for 4 h at room temperature (i.e., around 22°C–25°C).

### Antibody padlock probe (PLP) hybridization and ligation

**Timing: 2 h**

Unlike mRNA PLP, antibody PLP hybridization and ligation can be performed simultaneously in a single reaction. This section describes these steps.

18. Mix the reagent for antibody PLPs hybridization as the table shown below:

**Table:** 

	Reagent	Stock	Final	1× (μL)
1	NF-H_2_O	–	–	up to 30 μL
2	T4 Ligase buffer	10×	1×	3 μL
3	KCl	1 M	50 mM	1.5 μL
4	Formamide	100%	20%	6 μL
5	PLPs	1 μM/target	10 nM/target	0.3 μL/target
6	BSA	20 μg/μL	0.2 μg/μL	0.3 μL
7	T4 DNA Ligase	5 U/μL	0.5 U/μL	3 μL
	Total			30 μL/well

19. Wash the samples three times with 30 μL 2× SSC.
20. Add 30 μL of the solution made in step 18 to each sample.
21. Wrap the slide with Parafilm to prevent evaporation and aluminum foil prevented from light.
22. Incubate samples for 30 min at 37°C, then incubate for 90 min at 45°C.

### Rolling circle amplification (RCA)

**Timing: 16 h (overnight)**

This reaction will produce a long DNA amplification product containing repetitions of the complementary sequence of the PLP. The concentration of Phi29 enzyme and the reaction duration will influence the yield of this reaction. The different steps involved in RCA are described below.

23. Combine the reagents for RCA as shown in the table below:

**Table:** 

	Reagent	Stock	Final	1× (μL)
1	NF-H_2_O	–	–	up to 30 μL
2	10× Phi29 buffer	10×	1×	3 μL
3	Glycerol	50%	5%	3 μL
4	dNTPs (Deoxynucleotide triphosphates)	10 mM	0.25 mM	0.75 μL
5	BSA	20 μg/μL	0.2 μg/μL	0.3 μL
6	Exonuclease I	20 U/μL	0.2 U/μL	0.3 μL
7	RCA Primers	4 μM	200 nM	1.5 μL
8	Phi29 polymerase	10 U/μL	0.25 U/μL	0.75 μL
	Total			30 μL/well

24. Remove the PLPs ligation solution from each sample.
25. Wash the samples three times with 30 μL 2× SSC.
26. Add 30 μL of the solution prepared in step 23 to each sample.
27. Wrap the slide with Parafilm to prevent evaporation and aluminum foil prevented from light.
28. Incubate the samples at 37°C for 30 min, then 30°C and incubate overnight (about 16 h).

### Bridge probe (BrP) hybridization

**Timing: 1 h**

#### Day 3

This section describes the steps involved in BrP hybridization. The concentrations of the BrPs are essential for hybridization yield.

29. Make fresh 4× hybridization buffer (HB) every time:

**Table:** 

Stock solution	Volume	Final concentration
20× SSC	500 μL	4×
100% Formamide	500 μL	40%
NF-H_2_O	1 mL	–
Dextran sulfate (vortex to dissolve)	0.2 g	0.1%
Total	2 mL

30. Prepare the solution for BrP hybridization as below:

**Table:** 

	Reagent	Stock	Final	1× (μL)
1	NF-H_2_O	–	–	up to 30 μL
2	4× HB	4× SSC, 40% Formamide	2× SSC, 20% Formamide	15 μL
3	BrPs	10 μM/target	0.1 μM/target	0.3 μL/target
	Total			30 μL/well

31. Wash samples twice with 30 μL 2× SSC.
32. Remove the RCA solution from the samples.
33. Add 30 μL of the BrP hybridization solution prepared in step 30 to the sample.
34. Wrap the slide with Parafilm to prevent evaporation and aluminum foil prevented from light.
35. Incubate the samples at room temperature (i.e., around 22°C–25°C) for 2 h on a rocker (50 cycles per minute).

**CRITICAL:** We have optimized this step by using different concentrations of BrPs (50 nM and 100 nM). The higher concentration of BrP resulted in more spots (Figure 8).

### Readout probe (RoP) hybridization

**Timing: 1 h**

The steps below describe the RoP hybridization. The concentrations of the RoPs are essential for the yield of the hybridization.

36. Remove the BrP hybridization solution.
37. Wash the samples three times with 2× SSC.
38. Post-fixation after the BrP hybridization:
  a. Remove the 2× SSC buffer from the samples.
  b. Add 30 μL of 4% PFA solution to each sample.
  c. Incubate the samples for 10 min at room temperature (i.e., around 22°C–25°C).
  d. Remove the fixation solution from the samples.
  e. Wash the samples three times with 30 μL 1× PBS.
39. Prepare the RoP hybridization solution as below during the post-fixation step (step 38):

**Table:** 

	Reagent	Stock	Final	1× (μL)
1	NF-H_2_O	–	–	up to 30 μL
2	4× HB	4× SSC, 40% Formamide	2× SSC, 20% Formamide	15 μL
3	RoPs	10 μM/target	0.1 μM/target	0.3 μL/target
4	DAPI	100 μg/μL	1 μg/μL	0.3 μL
	Total			30 μL

40. Remove the washing buffer from the sample.
41. Add 30 μL of readout probe hybridization solution made in step 39 to each sample.
42. Wrap the slide with Parafilm to prevent evaporation and aluminum foil prevented from light.
43. Incubate for 1 h at room temperature (i.e., around 22°C–25°C), on a rocker.

***Note:*** Post-fixation with 4% PFA for 10 min is necessary if the sequential hybridization will be performed after the image acquisition of the first-round hybridization.

**CRITICAL:** We have optimized this step by using different concentrations of RoPs (10 nM, 20 nM, 40 nM, 60 nM, 80 nM and 100 nM). The higher concentration of RoPs resulted in more detected spots (Figure 8).

### Image acquisition

The below steps describe the imaging of the samples prepared in the previous steps. The specific optics, lasers, and model of the microscopes will influence how the parameters are determined.

44. Wash the samples three times with 30 μL 2× SSC.
45. Add 30 μL photo-protective buffer to each sample. The photo-protective buffer contains 50% glycerol, 75 μg/mL glucose oxidase, 520 μg/mL catalase and 0.5 mg/mL Trolox.
46. Images of sequential hybridization were acquired with a spinning disk confocal microscope (OLYMPUS, PlanXApo 60×/1.40- numerical aperture (N.A.) oil-immersion objective, and Andor iXon camera). DAPI was excited by 405 nm, and ATTO 488, TAMRA, CY5 was excited by 475 nm, 540 nm and 638 nm lasers respectively. The distance of Z-stacks was set as 0.5 μm. The exposure time and laser intensity were set differently in different channels to obtain the same maximum intensity value of spots.

**CRITICAL:** Increasing laser power or exposure time should be a first quick check when detecting a target for the first time.

***Note:*** We aligned the images from different hybridization rounds based on the DAPI images, as previous reported.^3^ More details about this is described in the supplementary section “Computational pipeline development of ARTseq-FISH” in our original publication.^1^ It is also possible to register the coordinates with TetraSpeck beads (T7279, Thermo Fisher Scientific).^3^

### Sequential hybridization

This section describes the protocol of stripping off the RoPs, sequential hybridization of a new set of RoPs, and acquiring images.

47. Incubate the samples with 30 μL 55%–65% formamide for 5–15 min.
48. Wash the samples three times with 30 μL 2× SSC.
49. Add 30 μL of a new set of RoPs solution prepared as shown in step 39 to each sample.
50. Incubate for 1 h and prevent from the light during the incubation.
51. Wash the samples three times with 30 μL 2× SSC.
52. Repeat steps 47–51 for four times (i.e., five rounds of imaging).

***Note:*** The signals obtained from ARTseq-FISH can also be detected with a wide-field microscope, making this technique more accessible.

**CRITICAL:** We do not recommend pausing once the protocol has started.

***Note:*** It is recommended to image the samples as soon as possible. However, if the samples are wrapped with Parafilm and aluminum foil and stored at 4°C, the signal can be detected even after a couple of weeks. Image acquisition for sequential hybridization should be performed in a single session. Do not acquire images from different rounds of hybridization on different days.

### Flow cytometry

This part provides an optional alternative measurement of mRNAs and (phospho-)proteins detected by ARTseq-FISH.

53. If the samples will be measured via flow cytometer, then the cells should be detached by trypsin before fixation.

***Note:*** Use Eppendorf tube to collect samples with 1 mL fixation and permeabilization solution. Blocking buffer, antibodies mix and washing solutions are 200 μL per tube. The reaction solution in the step 6, 12, 18, 23, 30, 39 mentioned above is 150 μL per tube. Place samples on a shaker (50 cycles/min) for all the steps.

**CRITICAL:** All the washing steps in the previous procedures should be performed with centrifugation. To prevent cell adhesion to the surface of Eppendorf tubes, it is advisable to use a 0.2 mg/mL BSA solution in PBS in all the washing steps.

**Figure 4:**
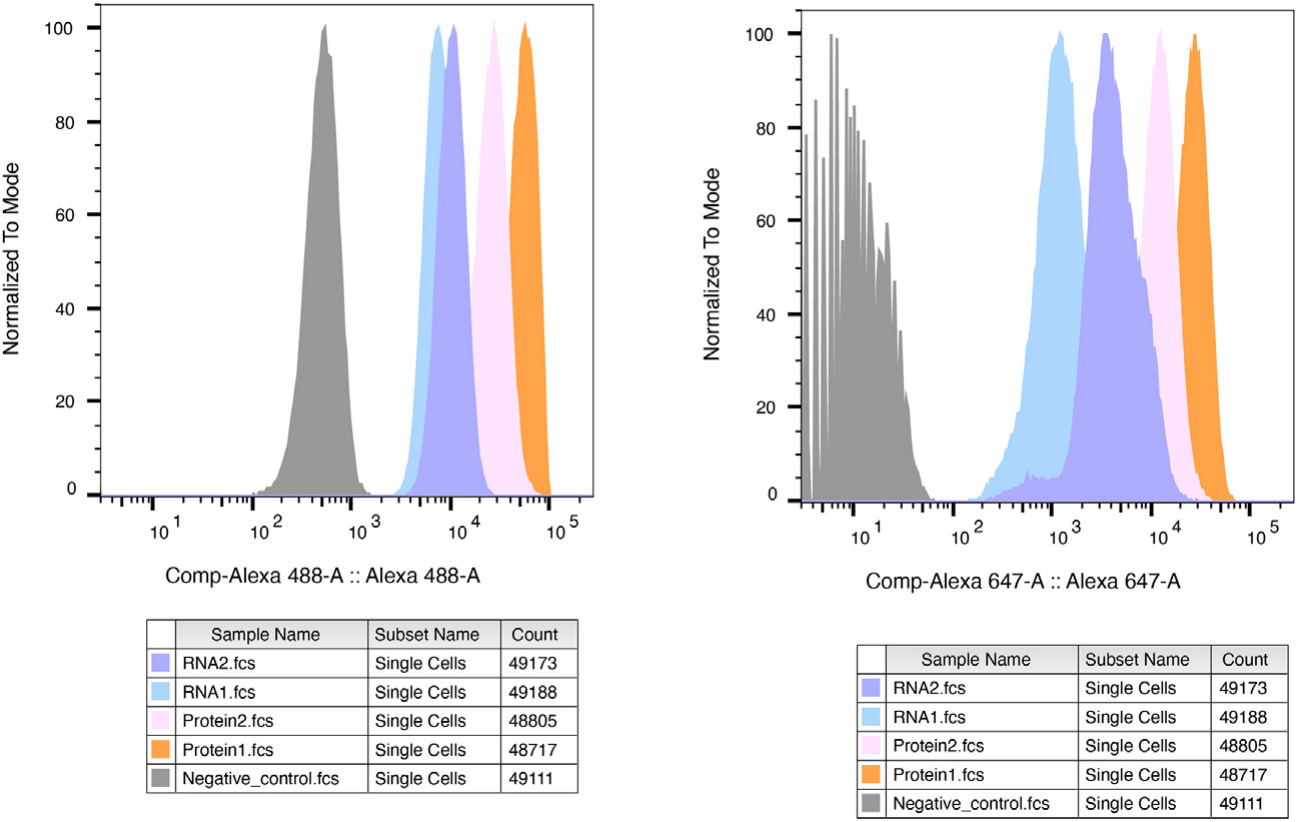
Expected measurement of the ARTseq-FISH results via flow cytometry Histogram showing the fluorescence intensity of two mRNA targets and two protein targets detected by ARTseq-FISH. Each target was read out in both Alexa 488 channel and Alexa 647 channel with two different RoPs. The detected intensity of all four targets shows the same trends in Alexa 488 channel and Alexa 647 channel. Negative control represents the sample without RoPs.

54. Measure the sample after the Step 1- 43 as shown previously with appropriate fluorescence lasers and filters as per fluorophore of readout probe.
55. Analyze the fluorescent signal from ARTseq-FISH probes using an Alexa Fluor 488 (488 nm) and Alexa Fluor 647 (627–640 nm) laser, 517/493 and 669/53 filter (Figure 4).

**CRITICAL:** It is not possible to achieve multiplexed detection by flow cytometry because the loss of localization information makes downstream decoding impossible.

## Expected outcomes

**Figure 3:**
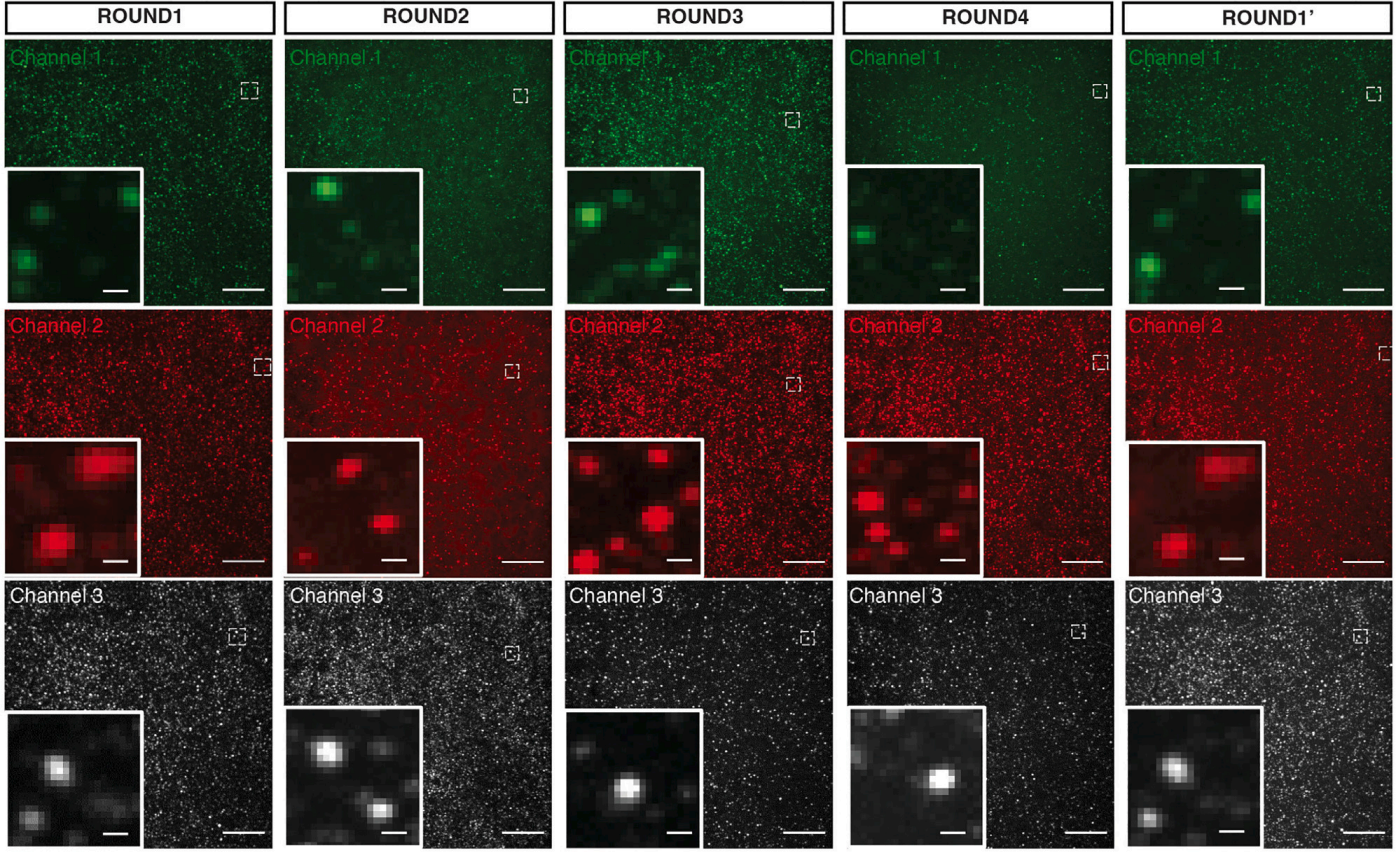
Simultaneous detection of 64 targets by ARTseq-FISH Representative z-projection images of detected mRNAs and (phospho-)proteins in 5 different hybridization rounds. Each channel contains multiple targets in each hybridization rounds. The 5th round (ROUND1′) detected the same targets as in the 1st round (ROUND1). Scale bar, 20 μm and 1 μm. Figure reprinted and adapted from Supplementary Figure 14 of Hu et al.^1^

**Figure 5:**
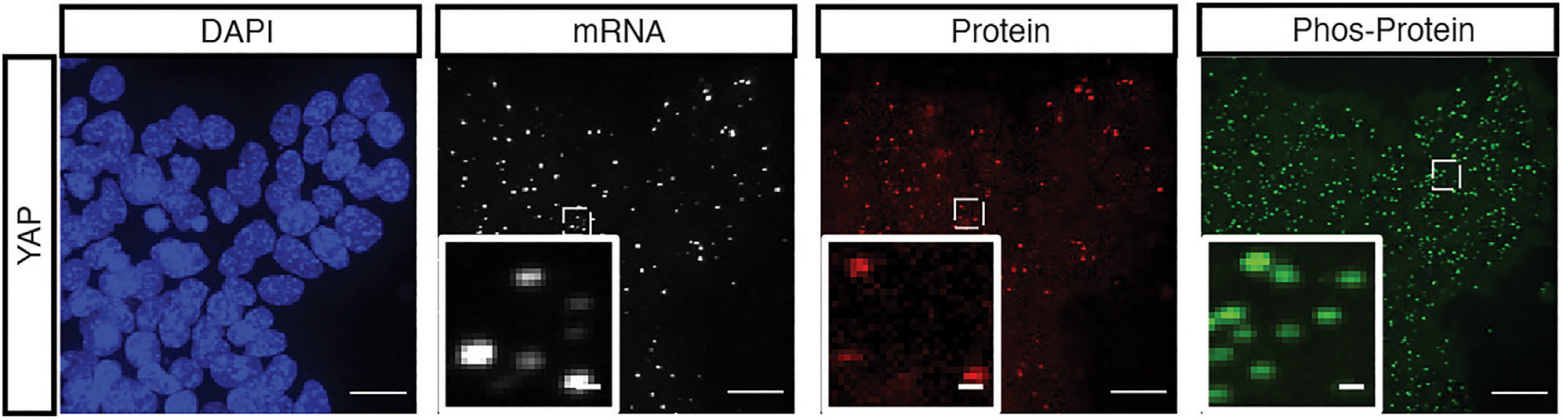
Simultaneous detection of mRNA and protein by ARTseq-FISH Representative z-projection images showing the detected YAP mRNA, YAP protein and phospho-YAP by ARTseq-FISH. DAPI shows the nuclei staining. Scale bar, 20 μm, 5 μm.

The protocol can simultaneously multiplex the detection of selected proteins and mRNAs at the same resolution (Figures 3, Figure 4 and Figure 5). This protocol can also be applied to simultaneously detect the mRNA, protein, and phosphorylated protein of a single target (Figure 5). The intensity of the spots did not change much; however, the number of the spots decrease a bit in the combined protocol. ARTseq-FISH results can also be measured by flow cytometry (Figure 4).

## Quantification and statistical analysis

We do not include the quantification, statistical analysis and decoding. The software for image analysis can be found at Hansen-Labs GitHub at http://github.com/Hansen-Labs/ARTseqFISH, code archived, see archive https://doi.org/10.5281/zenodo.10723692 or github.com/Hansen-Labs/ARTseqFISH. For more detail, refer to the Supplementary Information of the previous study.^1^

## Limitations

ARTseq-FISH relies on a series of probes to detect both mRNAs and proteins, therefore, the design of probes is essential for the detection.

There is risk that the selected probes are not specific or have low binding efficiency (especially for mRNA targets). There are methods using for instance 5 PLPs for one mRNA target,^7^^,^^10^ which increases the robustness of the signals; however, it is also more expensive and time-consuming. More importantly, more PLPs for one mRNA target generate bigger spots of detected mRNAs than detected proteins, which makes the image analysis more difficult. Using one padlock probe per mRNA provides the same detected resolution for mRNAs and proteins, but the probes need to be tested to know their performance in the experiments. We recommend detecting all the targets individually before applying their conjugated antibodies and probes in the final panel.

Another efficiency limitation in this method is the ligation reaction, i.e., mRNA PLP. The ligation of chimeric sequences with both DNA and RNA is challenging.^10^ The choice of ligase and ligation buffer is optimized in this protocol to increase the efficiency.

Although this technique has the potential to detect DNA (i.e., single nucleotide polymorphisms (SNPs)),^11^ miRNAs,^12^^,^^13^ viral RNA,^14^ and proximity of proteins^15^^,^^16^ or even proximity of RNA and protein, it is unable to detect double strand RNA and antisense RNA.

This protocol is optimized for mESCs, and some validation experiments were performed on MCF7, 3T3 cell lines. Additional optimization is needed for thick sections or paraffin embedded tissue sections. Lastly, imaging of sequential hybridization was performed manually in this protocol. An automated and robust imaging flow system will be helpful for multiplexing the detection.

## Troubleshooting

### Problem 1

**Figure 6:**
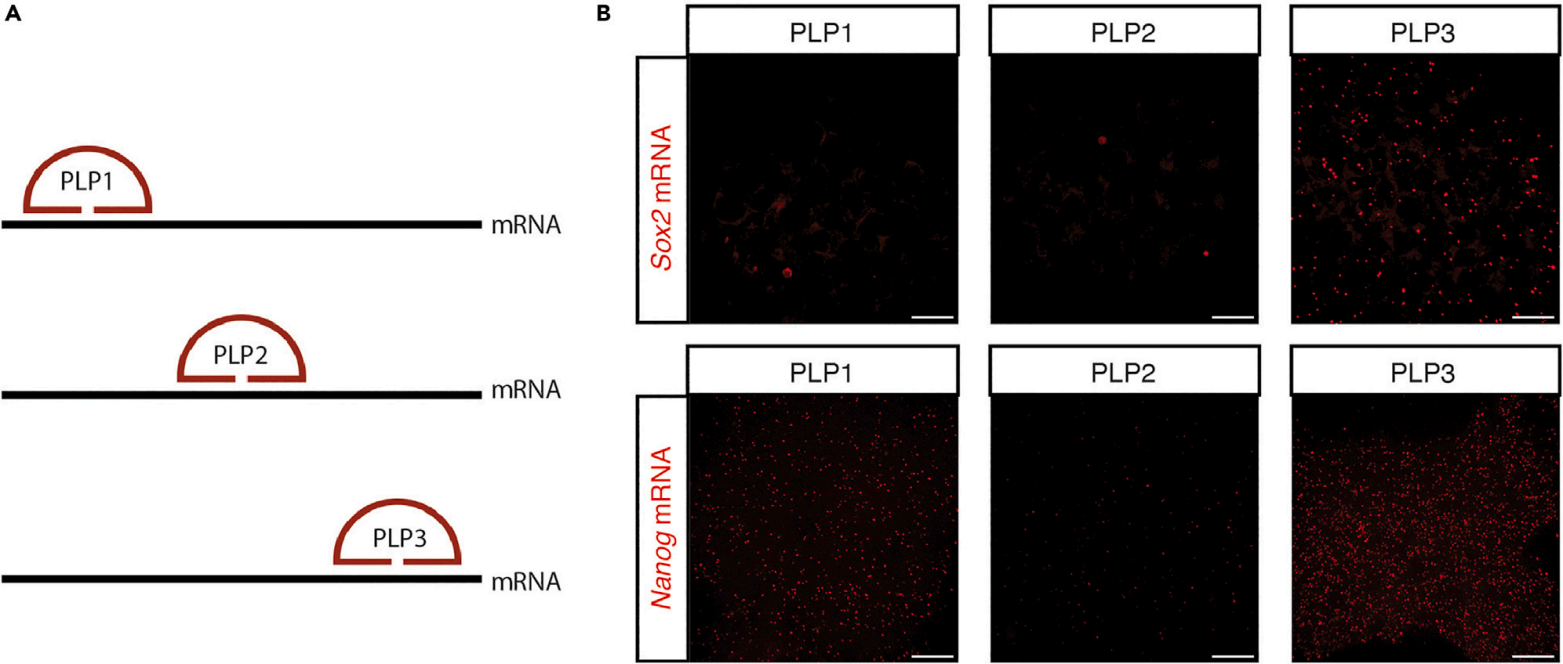
Different padlock probes targeting on different regions on mRNA result different detection efficiencies (A) Schematics showing the PLPs targeting on the different regions of mRNA targets. (B) Representative z-projection images showing the detection efficiency of the same target while using different PLPs. Scale bar, 20 μm. Figure reprinted and adapted from Supplementary Figure 10 of Hu et al.^1^

No signals for mRNA targets. If the mRNA was not detected with one PLP, the problem could be in the targeted sequence by the PLP of choice. As shown in the Figure 6, we designed three PLPs for *Sox2* and *Nanog* mRNA with different targeting regions on their mRNA sequences. Two PLPs of *Sox2* did not show positive signals comparing to the third one. Although all three PLPs of *Nanog* detected signals, the detection efficiency is variable.

### Potential solution

• Design different PLPs to target different regions of the mRNA target. See step 2 in Probe design.
• Five PLPs per mRNA target is generally recommended.^10^

### Problem 2

The number of spots for a particular target is less than expected.

### Potential solution

We optimized several steps by performing the reactions under different conditions, with different reagents or different concentrations of reagents.

**Figure 7:**
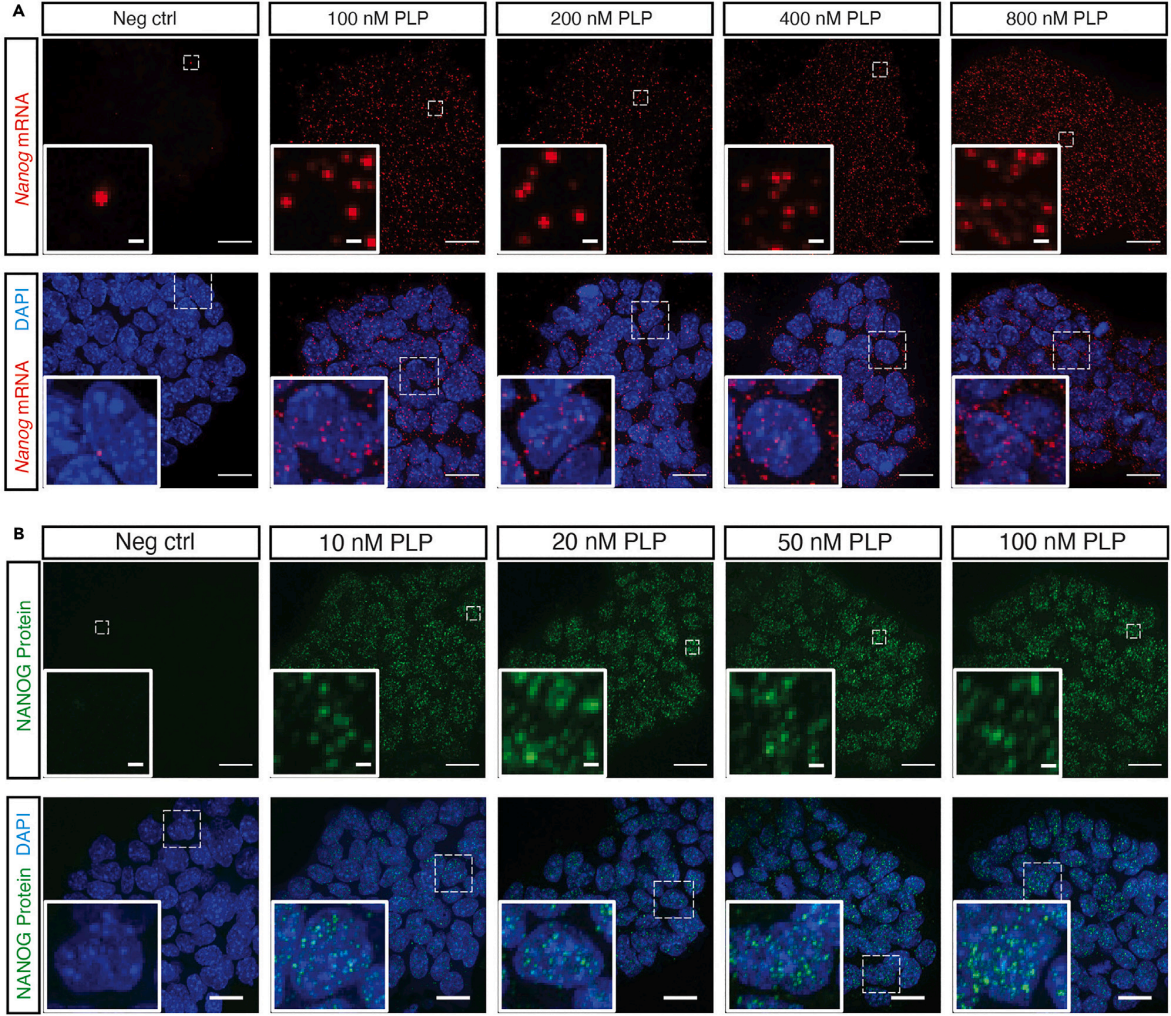
Different concentrations of padlock probes result in different detection efficiencies Representative z-projection images showing both the mRNA (A), and protein (B) detection efficiency are correlated with the concentrations of PLPs. Scale bar, 20 μm and 1 μm. Figure reprinted and adapted from Supplementary Figures 6 and 7 of Hu et al.^1^
• The detection efficiency is also correlated with the concentrations of BrPs (Figure 8B) and RoPs (Figure 8A). Increased concentrations of BrPs and RoPs will detect more spots per cell. We chose the highest concentrations of BrPs and RoPs we have tested; therefore, it is possible to use even higher concentrations of BrPs and RoPs to get more counts. See step 30 in bridge probe (BrP) hybridization and step 39 in readout probe (RoP) hybridization.

• The detection efficiency correlates with the concentrations of PLPs (both mRNA and protein) (Figure 7). Increased concentration of PLPs (both mRNA and protein) will result in higher detection yield of spots per cell. We chose the lowest concentration of PLP in our study, because we sought to examine the relative counts of each target and compared the changes of the relative counts over time during the exit of pluripotency. In addition, this also avoids optical crowding and reduces the cost. See step 6 in mRNA padlock probe (PLP) hybridization and step 18 in antibody padlock probe (PLP) hybridization and ligation.

**Figure 8:**
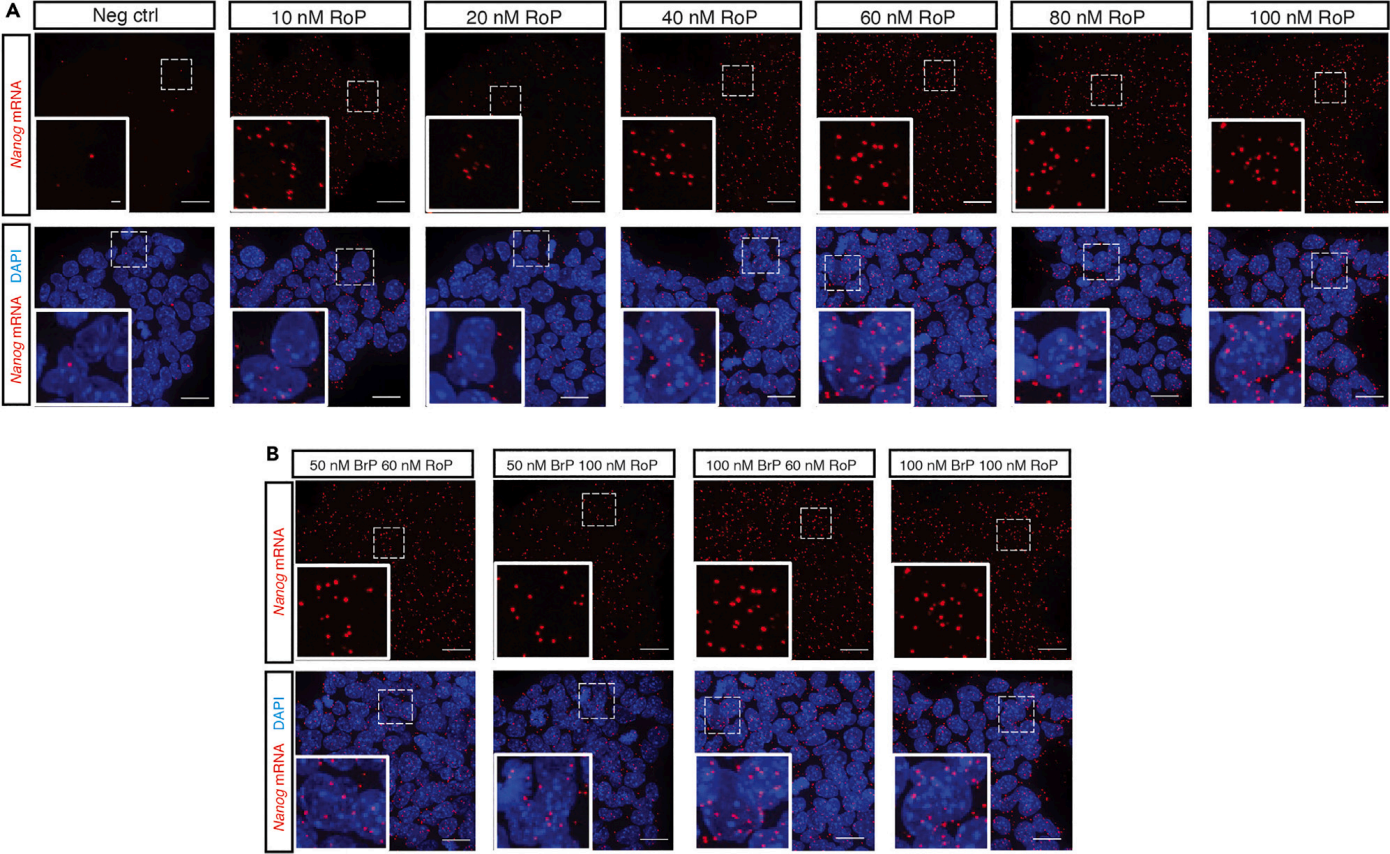
Different concentrations of bridge probes and readout probes result in different detection efficiencies Representative z-projection images showing both the detection efficiency is correlated with the concentrations of RoPs (A) and BrPs (B). Scale bar, 20 μm and 1 μm. Figure reprinted and adapted from Supplementary Figure 9 of Hu et al.^1^
• The duration and concentration of Phi29 polymerase could also influence the counts (Figure 9). We used the Phi29 polymerase from NEB, if use other brand or homemade Phi29 polymerase, the concentration of this enzyme could be adjusted to find a better working concentration. See step 23 in rolling circle amplification (RCA).

**Figure 9:**
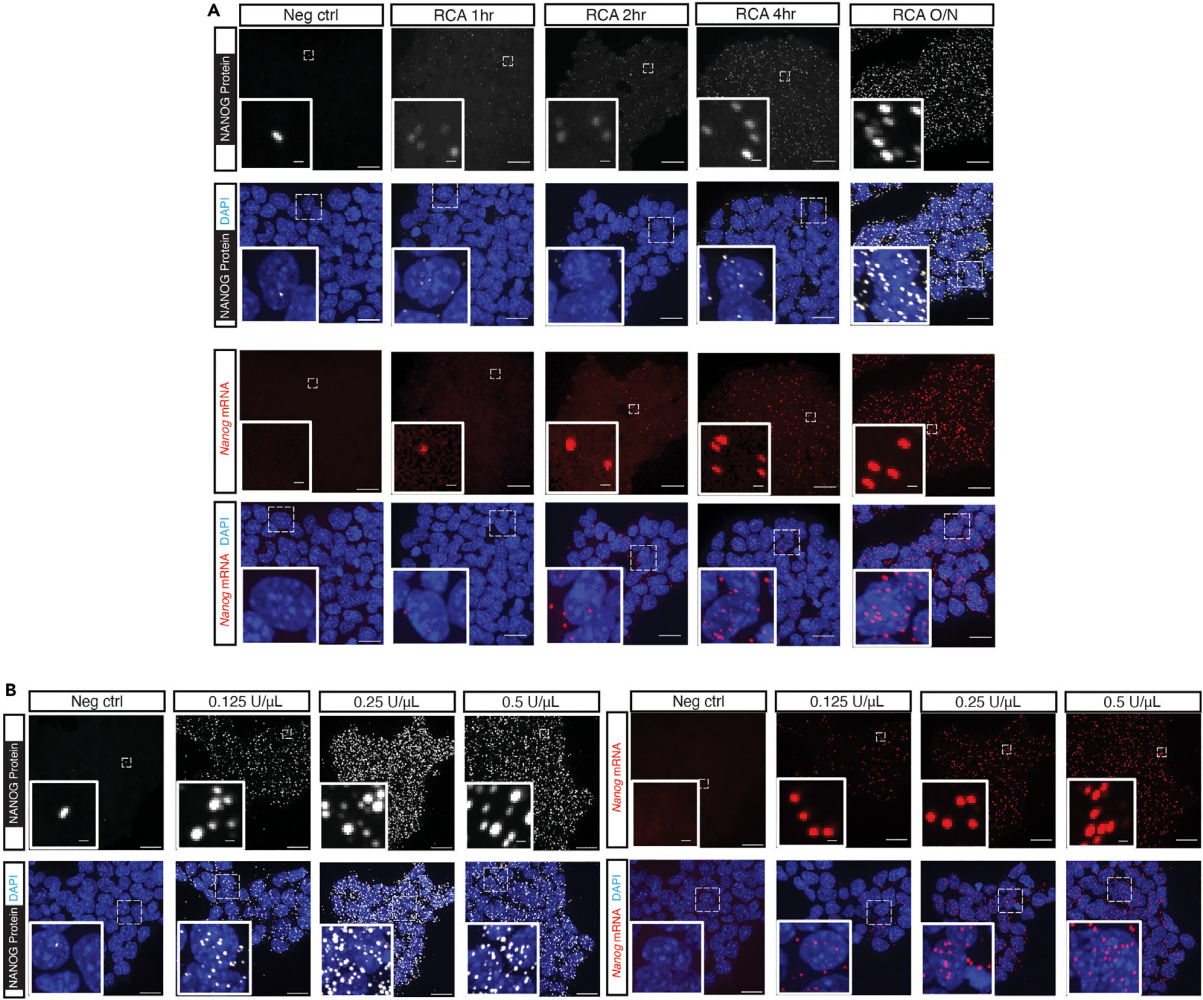
Different conditions of RCA result in different detection efficiencies Representative z-projection images showing both the incubation time (A) and the concentrations of phi29 (B) are correlated with the detection efficiency. Scale bar, 20 μm and 1 μm. Figure reprinted and adapted from Supplementary Figure 8 of Hu et al.^1^

### Problem 3

After removing the readout probe, fewer spots are detected in the sequential hybridization with the same readout probes.

### Potential solution

• Add a post-fixation step after BrP hybridization as suggested in the step-by-step protocol. See step 38 in readout probe (RoP) hybridization.
• Optimize the concentration of formamide and the incubation time of formamide. Different sequences of readout probes may need different incubation times to be removed. See step 47 in sequential hybridization.

### Problem 4

The detected spots are too crowded in one channel during the sequential FISH; therefore, it is difficult to decode the spots.

### Potential solution

• The optical crowding issue is mainly caused by protein detection. Diluting antibodies could be one option if relative counts are being measured. See step 5 in blocking and primary antibodies incubation.
• Assign to the same channel of one hybridization round, targets that are expected to be highly expressed together with those that are expected to be lowly expressed targets. See probe design.
• Assign fewer targets to be simultaneously detected in one channel in one hybridization round.

## Resource availability

### Lead contact

Maike M. K. Hansen (maike.hansen@ru.nl).

### Technical contact

Xinyu Hu (xinyu.hu@ru.nl).

### Materials availability

This study did not generate new unique reagents.

### Data and code availability

All relevant data supporting the key findings of this study are available within the research article and its Supplementary Information files.^1^ Sample images to test software are provided together with software, see archive https://doi.org/10.5281/zenodo.10723692 and github.com/Hansen-Labs/ARTseq-FISH. Raw imaging data is stored on a local drive. Original data have been deposited to both the Github repository and archived on Zenodo as 'Source data (publication).xlsx'. The data used to train the SVM models which detects nuclei and the point spread functions of hybridized signals can also be found in the archive. Figures 3, 6, 7, 8, and 9 are taken and adapted from supplementary figures in Hu et al.^1^

## Acknowledgments

We thank members of the Huck and Hansen laboratories for the thoughtful discussion and suggestions; Aigars Piruska for technical assistance with the spinning disk confocal; and Erik van Buijtenen and Kinga Matuła for sharing the antibody conjugation protocols, and fruitful discussions and suggestions from Erik van Buijtenen. We also thank Hendrik Marks for the mESC-E14. We acknowledge generous support from 10.13039/501100001832Radboud University (W.T.S.H.), the Christine Mohrmann fellowship (M.M.K.H.), the 10.13039/100011102European Union (ERC, ChOICE, 101041939, M.M.K.H.), the 10.13039/501100003246Netherlands Organization for Scientific Research (NWO) (Spinoza grant, W.T.S.H.), and 10.13039/501100021821Oncode Institute, which is partly financed by the 10.13039/501100004622Dutch Cancer Society (M.M.K.H.). X.H. acknowledges financial support from the China Scholarship Council (CSC).

## Author contributions

X.H., B.v.S., W.T.S.H., and M.M.K.H. conceived and designed the study. X.H. developed and optimized the experimental procedure for ARTseq-FISH. B.v.S. developed the software for ARTseq-FISH. Ó.G.-B. helped with probe design and optimization of ARTseq-FISH workflow. X.H. wrote the manuscript, and Ó.G.-B., B.v.S., W.T.S.H., and M.M.K.H. edited the manuscript.

## Declaration of interests

The authors declare no competing interests.
